# Moral grandstanding in public discourse: Status-seeking motives as a potential explanatory mechanism in predicting conflict

**DOI:** 10.1371/journal.pone.0223749

**Published:** 2019-10-16

**Authors:** Joshua B. Grubbs, Brandon Warmke, Justin Tosi, A. Shanti James, W. Keith Campbell

**Affiliations:** 1 Department of Psychology, Bowling Green State University, Bowling Green, Ohio, United States of America; 2 Department of Philosophy, Bowling Green State University, Bowling Green, Ohio, United States of America; 3 Department of Philosophy, Texas Tech University, Lubbock, Texas, United States of America; 4 Department of Psychology, University of Georgia, Athens, Georgia, United States of America; Valparaiso University, UNITED STATES

## Abstract

Public discourse is often caustic and conflict-filled. This trend seems to be particularly evident when the content of such discourse is around moral issues (broadly defined) and when the discourse occurs on social media. Several explanatory mechanisms for such conflict have been explored in recent psychological and social-science literatures. The present work sought to examine a potentially novel explanatory mechanism defined in philosophical literature: Moral Grandstanding. According to philosophical accounts, Moral Grandstanding is the use of moral talk to seek social status. For the present work, we conducted six studies, using two undergraduate samples (Study 1, *N* = 361; Study 2, *N* = 356); a sample matched to U.S. norms for age, gender, race, income, Census region (Study 3, *N* = 1,063); a YouGov sample matched to U.S. demographic norms (Study 4, *N* = 2,000); and a brief, one-month longitudinal study of Mechanical Turk workers in the U.S. (Study 5, Baseline *N* = 499, follow-up *n* = 296), and a large, one-week YouGov sample matched to U.S. demographic norms (Baseline *N* = 2,519, follow-up *n* = 1,776). Across studies, we found initial support for the validity of Moral Grandstanding as a construct. Specifically, moral grandstanding motivation was associated with status-seeking personality traits, as well as greater political and moral conflict in daily life.

## Introduction

Public discourse is often fraught with controversy, particularly when the focus of such discourse is related to issues of morality or political belief [1]. Value-based discussions are prone to conflict, as individuals engaged in such conversations are often passionately invested in their beliefs and positions [2–5]. A body of research has noted that, particularly in the context of social media environments and electronic communication methods, public discussion of controversial topics has become even more volatile over recent years [6–9]. A number of reasons for such conflicts have been suggested in recent literatures, many of which have shown great promise in describing or predicting caustic forms of dialogue. Yet, even with such advances, there are still many aspects of public discourse that are poorly understood or relatively unstudied in contemporary psychological literature. To address this need, the purpose of the present work is to examine one factor that might be particularly relevant to ongoing studies of social conflict in public discourse about morally charged topics: moral grandstanding [10]. Adapted from philosophical literature [i.e., 10], moral grandstanding refers to the use of moral talk to seek social status. Heretofore unexamined by behavioral science, the present work seeks to examine if moral grandstanding might be relevant for understanding at least some part of current conflicts in public discourse.

### Moral discourse and social media

In recent years, a number of studies have documented the socially caustic nature of public discourse, particularly on social media [11]. Social media users are known to increasingly self-select into homogenous social groups [12–15], which may flow from a more innate tendency in humans to be inwardly focused and in-group biased [16]. Building on this tendency toward self-segregation, other work has demonstrated that social media has exacerbated natural human tendencies toward in-group/out-group biases [17,18]. Ultimately, this seems to have contributed to “echo chamber” environments, where ideological positions are continuously reinforced, with little exposure to other ideas [12,18,19]. Moreover, it seems that, when people are actually exposed to opposing viewpoint, this exposure often actually increases polarization [20].

Affective and ideological polarization are well-documented [20,21], and perceived polarization (i.e., the belief that opposite sides in an argument are extremely far apart) seems to outpace actual polarization [22–24]. In documenting these trends, many have noted that social media itself seems to be a driving factor in the experience of polarization and conflict-ridden dialogue [21]. That is, the always-on nature of social media provides opportunities for social conflict in a quantity and consistency that did not exist before the internet [25], and may be responsible for documented trends in polarization and discord.

Despite the above findings, however, it is not accurate to conclude that there is universal consensus regarding the role of social media in driving dysfunctions in public-discourse. Specifically, some work has suggested that polarization is occurring more rapidly among those who use the internet less [26]. Such a finding suggests that polarization may be attributable to multiple factors, rather than solely to social media or digital online environments themselves. However, there is little disagreement with the notion that public discourse on social media platforms is particularly prone to conflict and outrage [27,28]. That is, moral outrage in the digital age is common and often especially intense [1]. Moreover, the prevalence of such outrage is particularly concerning given research showing that outrage occurring on social media is often quickly realized but imprecisely pointed at diverse potential targets [1,7].

Collectively, empirical research suggests that online environments—particularly social media—are often full of caustic examples of public discourse. Several mechanisms may be at play, including natural tendencies toward homogeneity, ingroup/outgroup biases, and a culture that encourages outrage. However, another possible factor that has been heretofore understudied as a potential driving mechanism in polarization and outrage in social discourse is the inherent human drive to seek status or rank. That is, public discourse, particularly on social media, is often directly or indirectly concerned with increasing one’s influence, rank, or social standing [29–31], which may be relevant to understanding why such discourse is often caustic and conflict prone.

### Status-seeking

Humans are inherently social animals with well-documented tendencies to seek affiliation, social bonding, and community identity [32]. However, humans are also driven to seek status within their communities and spheres of belonging [33–35]. In the context of human evolution, high social status was most likely advantageous in mate selection and resource competition, which likely accounts for why human beings crave influence and social power [36–38]. Building on this innate tendency, there is now considerable evidence that humans seem to be predisposed toward two primary avenues of seeking status: dominance and prestige [33].

Dominance refers to the tendency to seek social status and influence (and the associated resources and advantages associated with such influence) by means of dominating, controlling, or subduing opponents [39]. Although dominance can certainly be achieved through physical means (i.e., violence), humans are particularly adept at using social cues, verbal behavior, non-verbal behavior, and interpersonal dynamics to achieve dominance without physical coercion [40–42]. For example, a person may use verbal threats or attempt to shame or embarrass an ideological opponent or rival, as a means of seeking social status via dominance.

Like dominance, prestige is similarly concerned with status and influence seeking, but seeks to do so through less caustic means. That is, prestige specifically refers to attempts to seek status or influence via gaining the respect and admiration of others [33,43]. Whereas dominance involves coercing others into submission, prestige is often more inspirational in nature. Prestige may be characterized by a desire to be recognized for skills, “know-how,” or general abilities.

In social settings, both dominance and prestige are viable options for upward social mobility [33,43]. Although dominance often comes at some social cost (e.g., likability, perceived agreeableness), it is often useful in attaining status and rank. Prestige is similarly effective for achieving such goals, often with the added benefit of enhancing likability and perceived agreeableness [34]. Even so, social status-seeking often entails conflict and competition, especially when multiple forces are seeking rank or status or when resources are scarce. Although dominance is a clear pathway to such conflict [44], prestige is not exempt from this consequence [39].

Although relatively unexamined in analyses of public discourse, it is likely that status-seeking motives are a consistent aspect of such discourse as well. As previously referenced, social media engagement and activity is often characterized by status-seeking motives [29–31]. Building on these premises, the present work seeks to examine if status-seeking motivations may be of relevance in understanding problems in public discourse in the modern era. To our knowledge, no prior work has specifically postulated that status-seeking via dominance and prestige may be a relevant mechanism in understanding political and moral polarization in public discourse.

### Moral grandstanding

Recently, moral philosophy has described the phenomena of status-seeking via moral discourse. Moral grandstanding (hereafter, MG) is the use of public moral discourse for self-promotion and status attainment [10]. According to Tosi and Warmke’s initial explanation on the topic, an individual engaging in MG is likely to use public discussion of morality and politics to impress others with their moral qualities. Importantly, as it is currently defined, MG is distinguished by what motivates it. That is, at its core, MG is defined by the motivation that underlies it. While there may be many reasons to engage in public discourse (i.e., to share one’s beliefs or to express moral opinions to others), MG specifically refers to engagement that is motivated to a significant degree by a desire to enhance one’s status or ranking. On this point, a helpful comparison can be made with lying. Lying is not defined simply by whether or not what a person says is false. Lying generally requires a desire or intention to deceive [e.g., 45]. Two people could say the exact same untrue sentence, but only one of them might be trying to deceive you, and only that person is lying. Similarly, what is crucial to understand about MG—as defined in philosophical literature—is that it is a contribution to public discourse that is motivated to a significant degree by the desire to impress others. This means that one cannot simply look at the meaning of what someone says and know whether it is grandstanding. Like other discursive phenomena, (e.g., lying or bragging), motivation is the key characteristic.

For example, in an effort to impress his friends with his saintly character, someone might say, “I have long stood on the side of the disadvantaged and this case is no exception. I will not tolerate this injustice, and I can’t believe that any decent person is seriously debating this issue. We must remember: the world is watching.” Some who grandstand want to be seen as moral exemplars. Others want to be seen as merely being on the “right side of history.” Those who engage in MG may be sincere when they make their ostentatious claims about any particular issue (e.g., justice, morality, human rights, or patriotism). In contrast, those who engage in insincere MG may not care one way or another about their moral stand, but want their audience to think they care deeply. MG takes many forms. In a quest to impress peers, someone may trump up moral charges, pile on in cases of public shaming, announce that anyone who disagrees with them is *obviously* wrong, or exaggerate emotional displays in taking ideological positions. They may also engage in a kind of moral one-upmanship, or “ramping up,” making increasingly strong claims to “outdo” other discussants as they try to show that they are more morally sensitive, or care more about justice. Ultimately, as conceptualized in philosophical literature, MG is likely to lead to conflict and difficulties in discourse around moral and political topics.

Returning to psychological literature, conceptually, MG is somewhat similar to previously documented constructs such as social vigilantism [27,28], which is concerned with a desire to correct the incorrect social views of others. This particular construct deserves special consideration, given that social vigilantism is specifically focused on public discourse, and that it seems to be associated with a willingness to share or promote one’s moral views. Indeed, past work has demonstrated that social vigilantism predicts engagement in social forums and at least partially accounts for the link between outrage and social media engagement displaying that outrage [46]. Importantly, however, in its original conception [27,28], social vigilantism was not explicitly linked to status-seeking drives and behaviors (though it was positively linked with narcissism). As such, in premise, MG motivation should be distinct from social vigilantism, in that its conceptual focus on status seeking, rather than on being correct or correcting others.

MG motivation may also have some similarities to prior attempts to measure concerns for political correctness [47], which measures personal motivations to correct politically incorrect speech. However, while concern for political correctness does involve moral speech in public discourse, it is not explicitly concerned with status-seeking motivations in the same way that MG is. Additionally, past evidence suggests that concern for political correctness is a somewhat partisan construct, being associated with more liberal values and lower levels of right-wing authoritarianism [47]. MG, however, as conceptualized, should be an ideologically neutral tendency. That is, the tendency to use moral speech for self-serving, status-seeking motivations is not likely attached to specific ideology, being more similar to an individual difference variable.

We also note that MG may also be similar to belief superiority, or the tendency to think that one’s specific beliefs are superior to the beliefs of others [48]. However, despite compelling evidence that belief superiority is an important construct of interest in understanding polarization in public discourse [27,48,49], prior works related to belief superiority have generally focused on the superiority of individual beliefs, rather than on more general motivations to use beliefs and moral talk to advance status. In contrast, in philosophical accounts, MG is associated more with a desire to be seen as someone to be respected or admired than it is with a desire to be correct or to have superior beliefs. In this way, MG is likely similar to narcissism more broadly, particularly grandiose narcissism which involves acclaim-seeking, status motivations, and a desire to be respected and admired by others [50].

Another potential construct that might be similar to MG is a phenomenon commonly referred to as “virtue signaling” [51]. Although somewhat neglected in psychological or social-science literature, “virtue signaling” seems most often to be used as a derogatory term directed at individuals whom one thinks are using moral or value-based public speech as a means of demonstrating their virtues or piety, typically with a goal of seeking status or respect in the eyes of likeminded others [52]. That is, virtue signaling, as commonly understood, refers publicly signaling one’s values or virtues in a grandiose way with a goal of garnering attention and admiration. Although such a term is, perhaps, colloquially useful to describe certain types of public discourse, we note that virtually no empirical literature exists examining “virtue signaling” as a discrete psychological construct, nor is there any substantive work articulating its nomological network or rational boundaries in philosophical inquiry. This stands in contrast to MG, which has been extensively explored and defined in philosophical literature. As such, it is our contention that MG is likely better-defined construct that is more suitable to exploration and adaptation as a psychological construct.

## The present study

Based on the above literature, the primary purpose of the present work was to attempt to define, assess, and describe MG as a psychological phenomenon, and to validate its use as a variable of interest in studying polarization and conflict in public discourse.

As defined in philosophical literature, MG is inherently related to status-seeking and defined by motivation rather than specific behaviors. As such, we defined it in terms of previous status-seeking literature in order to explore its relevance as a psychological construct. Specifically, we hypothesized that such a construct would likely emerge in ways consistent with previously reviewed literature on dominance and prestige as universal means of human status-seeking. Based on this expectation, we attempted to measure MG as it might be expressed in two dimensions: Dominance strivings and Prestige strivings.

Given that MG is, by definition, concerned with the motives that underlie public discourse activities, we elected to focus on assessing this phenomenon via self-reported motives for this preliminary study. That is, we considered the motivation to engage in MG to be an individual difference variable, rather than focusing on specific instances of behavior that may or may not be MG. Additionally, though we expected that MG Motivation likely has social and relational signifiers, we restricted the scope of this preliminary study to the foundation of the construct: individual motives and attitudes underlying public discourse activities. Hereafter, we use the term “MG Motivation” to refer to the construct as we measured it and just “MG” to refer to the actual behaviors described in philosophical accounts of the phenomenon.

As MG Motivation inherently involves seeking status and rank, we also speculated that it would be associated with personality traits associated with status and acclaim seeking tendencies, as well as tendencies toward social dominance [e.g., antagonistic tendencies 53–55]. To this end, we expected that MG Motivation would be positively associated with narcissistic antagonism (particularly to the extent that MG Motivation is expressed as dominance) and narcissistic extraversion (particularly to the extent that MG Motivation is expressed as prestige). Moreover, given the known positive associations between status-seeking and extraversion more generally and the known negative associations between status seeking and agreeableness [56], we expected to see similar patterns of associations between MG Motivation and basic personality traits (i.e., Big Five factors). Finally, given that philosophical accounts describe MG as a clear risk for conflict and dysfunction in public discourse, we predicted that MG Motivation would be associated with and predictive of greater conflict in daily life about moral or political topics, as discussions about such topics are the key domains in which MG is most likely to be salient. Finally, we sought to differentiate MG Motivations from similar constructs (i.e., status-seeking more broadly and social vigilantism).

To test the above ideas, a series of studies using diverse sampling methods and procedures were conducted. More information regarding the following studies are available via the Open Science Framework at: https://osf.io/r3j45/?view_only=33f13eba6615430296ac476fc8e61b50.

All studies were approved via the Bowling Green State University Institutional Review Board (BGSU IRB #’s: 1139296, 1315282, 1288212, 1233172, 1407492). All participants were presented with written electronic informed consent documents at the outset of each study and subsequently gave informed consent before engaging in these studies.

## Study 1

In order to construct and initially validate a measure of Moral Grandstanding Motivations, a cross-sectional study of undergraduate students was conducted.

### Participants and procedure

Participants were undergraduates at a large public university in the midwestern United States (*N* = 388; *M*_*age*_ = 18.99, *SD* = 1.55; 72.1% women). Sample size was determined by obtaining the largest possible sample in a single semester. The predominant race of participants was White/Caucasian (81.2%) followed by African-American/Black (14.5%), Latinx/Hispanic (4.3%), American Indian/Native American/Alaska Native (1.5%), Asian/Pacific Islander (1.3%), Middle Eastern (1.3%) and other (0.3%). Participants identified their political parties as being Democrat (29.4%), Republican (27.4%), No Party (27.7%), Independent (6.5%), Libertarian (6.5%%), and Other (2.5%).

Participants were offered partial course credit for their participation in psychology research activities, with this particular study being one of several options afforded during the Fall, 2017 semester.

### Measures

#### Personality

In order to control for relevant aspects of personality that may be key to understanding the phenomenon of MG, two measures were included. Primarily, we included the IPIP-NEO-120, which is a 120-item inventory of the Five Factor Model of personality developed from the International Personality Item Pool [57]. Although the IPIP provides six facet scores for each domain of personality, the focus herein was on the five main factors themselves (Openness, Conscientiousness, Extraversion, Agreeableness, and Neuroticism). Despite our focus on these aggregate scores, we also included all facet score associations as supplemental tables (S2A and S2B Table).

Additionally, we included the Five Factor Narcissism Inventory 60 [58], which is derived from the FFNI-120 [50,59,60]. Based on the Five Factor Model of Personality, the FFNI-60 measures numerous domains of narcissistic personality along several facets. For the present work, three composite indices were of focus: Narcissistic Antagonism (which measures the aggressive, exploitative, impulsive, and socially caustic aspects of narcissism), Narcissistic Extraversion (which measures the grandiose, gregarious, and outgoing aspects of narcissism), and Narcissistic Neuroticism (which measures the vulnerable, reactive aspects of narcissism). Despite our focus on these aggregate scores, we also included all facet score associations as supplemental tables.

#### Political affiliation

We asked participants to identify their political party of preference. Available responses included, “Democrat,” “Republican,” “Green Party,” “Libertarian,” “Tea Party,” “Independent,” “No party,” and “other.”

Additionally, we asked participants to rate their political orientation. Participants responded to the item, “Political views are often expressed in terms of right (conservative or traditional) vs. left (liberal or progressive), using the slider below, please indicate where you believe your political views to align” on a range of -10 to positive 10. For the purpose of this question, the midpoint (0) was the scale anchor.

#### Moral grandstanding motivation

MG Motivation was assessed using a bank of 29 items generated for the present study by a team of experts in psychology and philosophy (See S1 Table). This item bank was generated with an a priori goal of obtaining a final instrument of 10 items with two subscales assessing the previously two described dimensions of MG Motivation: Prestige Strivings (e.g., “My moral/political beliefs should be inspiring to others.”) and Dominance Strivings (e.g., “I share my moral/political beliefs to make people who disagree with me feel bad”). The complete list of items tested are available in the supplemental materials (S1 Table). Participants read the following prompt: “The following items refer to your political or moral beliefs. Take a moment to think about your strongest held moral and political beliefs. Think about the issues that are most important to you and the things that you are most passionate about. After thinking about these things, please answer the following questions.” Responses were coded on a scale of 1 (*strongly disagree*) to 7 (*strongly agree*).

#### Political/Moral conflict

Participants also reported the extent to which they had experienced political or moral conflict in daily life over the twelve months prior to the survey. Participants reported the frequency with which they had experienced 10 things (“lost friends because of my political/moral beliefs,” “gotten into debates on social media because of my political/moral beliefs,” “gotten into fights on social media because of my political/moral beliefs,” “grown distant from a friend because of my political/moral beliefs,” “experienced conflict in my home because of moral/political issues,” “felt as if moral/political issues were interfering with my relationships,” “gotten into fights on social media because of the political or moral beliefs of others,” “tried to convince someone that their political or moral beliefs were wrong,” “severed ties with a friend over moral/political differences,” and “ended a relationship with a family member over moral/political issues”). Responses were recorded on a scale of 1 (*never*/*not at all*) to 4 (*several times*). Responses were averaged.

### Analyses

In order to shorten the proposed Moral Grandstanding Motivation Scale (MGMS) into a two sub-scale, 10-item measure, we conducted a maximum likelihood factor analysis with Direct Oblimin rotation [61,62]. Items were selected based on factor loadings, factor cross-loadings, item readability, and redundancy. That is, items were retained both on the basis of statistical merits and conceptual clarity.

Upon reduction of the 29-item test bank into a two-factor, 10-item scale, both resultant scale scores were computed (averaged across items) and correlated with the aforementioned personality measures and conflict measures, to establish validity. We additionally compared respondents’ MG scores, based on political ideology (using ANOVAs).

Finally, we conducted a two-step hierarchical regression to establish the unique contribution of MG Motivation (step 2) to political/moral conflict, above and beyond basic personality factors and left-right political ideology (Step 1).

### Results

All descriptive statistics for Studies 1–5 are included in Table 1.

**Table 1:** Descriptive statistics for included variables.

		Study 1 (*N* = 361)		Study 2 (*N* = 356)		Study 3 (*N* = 1,063)		Study 4 (*N* = 2,000)		Study 5 (*N* = 499 [T2 = 296])	
	Pos. Range	M (SD)	α	M (SD)	α	M (SD)	α	M (SD)	α	M (SD)	α
FFNI Antagonism	1–5	2.39 (0.61)	.926	2.38 (0.58)	.911	2.25 (0.63)	.925	-	-	2.81 (1.05)	.977
FFNI Extraversion	1–5	3.39 (0.56)	.817	3.33 (0.65)	.851	2.96 (0.72)	.881	-	-	3.21 (0.86)	.922
FFNI Neuroticism	1–5	3.16 (0.74)	.875	3.22 (0.77)	.872	2.87 (0.76)	.857	-	-	3.00 (0.70)	.812
Openness	1–5	3.22 (0.50)	.824	3.25 (0.50)	.800	3.16 (0.50)	.805	-	-	3.13 (0.63)	.865
Conscientiousness	1–5	3.59 (0.55)	.890	3.55 (0.55)	.888	3.78 (0.62)	.917	-	-	3.55 (0.64)	.897
Extraversion	1–5	3.50 (0.57)	.895	3.46 (0.59)	.890	3.21 (0.63)	.901	-	-	3.34 (0.71)	.918
Agreeableness	1–5	3.65 (0.54)	.882	3.61 (0.49)	.842	3.72 (0.54)	.865	-	-	3.41 (0.67)	.889
Neuroticism	1–5	3.01 (0.59)	.882	3.07 (0.63)	.891	2.65 (0.76)	.929	-	-	2.85 (0.76)	.919
Political Spectrum*	-10–10	-0.09 (5.48)	-	-0.57 (5.50)	-	0.74 (5.80)	-	3.01 (1.23)^†^	-	-	-
Right Wing Authoritarianism		-	-	-	-	3.52 (1.21)	.937	-	-	-	-
Left Wing Authoritarianism		-	-	-	-	3.61 (0.75)	.811	-	-	-	-
**Moral Grandstanding: Prestige Strivings**	**1–7**	**4.08 (1.16)**	**.817**	**3.99 (1.28)**	**.814**	**4.30 (1.25)**	**.843**	**4.61 (1.15)**	**.837**	**4.53 (1.17)** **[T2: 4.44 (1.11)]**	**.855** **[.814]**
**Moral Grandstanding: Dominance Strivings**	**1–7**	**2.58 (1.32)**	**.878**	**2.39 (1.33)**	**.894**	**2.40 (1.44)**	**.912**	**2.45 (1.39)**	**.902**	**2.43 (1.57)** **[T2: 2.19 (1.29)]**	**.952** **[.905]**
Political/Moral Conflict	1–4	1.49 (0.58)	.911	1.53 (0.62)	.905	1.51 (0.66)	.903	-	-	1.54 (1.25) [T2: 1.36 (0.55)]	.925 (.904)

*-10 corresponded to “left” political/moral views and 10 corresponded to “right” political/moral views;

^†^For Study 4, Political spectrum was measured via YouGov’s standard 7-point ideology question (1 = Strongly liberal, 2 = Somewhat Liberal, 3 = Moderate, 4 = Somewhat Conservative, 5 = Strongly Conservative);

The specified-two factor solution accounted for 46.64% of the variance in the total model (See S1 Table). Inspection of the scree plot similarly supported the specified two-factor solution (See S1 Fig). Based on factor loadings, item clarity, and reducing redundancy, the scale was restricted to 10 items: 6 assessing a desire to inspire others and gain respect and 4 assessing a desire to shame or silence others. These items, their means, standard-deviations, skewness, and factor loadings are available in Table 2. The resulting scale was titled, the Moral Grandstanding Motivation Scale (MGMS), with subscales that were subsequently titled Prestige Strivings (MGMS:PS) and Dominance Strivings (MGMS:DS).

**Table 2:** Confirmatory factor loadings for the Moral Grandstanding Motivation Scale.

Item	Study 2			Study 3			Study 4			Study 5		
	*M (SD)*	MGM: Prestige	MGM: Dominance	*M (SD)*	MGM: Prestige	MGM: Dominance	*M (SD)*	MGM: Prestige	MGM: Dominance	*M (SD)*	MGM: Prestige	MGM: Dominance
**1**.	4.27 (1.54)	.668	-	4.56 (1.5)	.713	-	4.82 (1.49)	.698	-	5.03 (1.45)	.695	-
**2**.	3.73 (1.67)	.616	-	4.16 (1.63)	.702	-	4.39 (1.55)	.691	-	4.81 (1.57)	.753	-
**3**.	3.9 (1.56)	.742	-	4.23 (1.5)	.741	-	4.59 (1.46)	.736	-	4.87 (1.48)	.764	-
**4**.	4.07 (1.69)	.758	-	4.23 (1.68)	.747	-	4.43 (1.62)	.713	-	4.77 (1.65)	.786	-
**5**.	4.36 (1.53)	-	830	4.73 (1.5)	-	.842	2.37 (1.55)	-	.785	3.37 (2.1)	-	.897
**6**.	3.72 (1.61)	-	.855	4.2 (1.62)	-	.843	2.42 (1.56)	-	.836	3.4 (2.11)	-	.922
**7**.	2.38 (1.51)	-	.748	2.39 (1.6)	-	.852	2.39 (1.59)	-	.840	3.39 (2.12)	-	.925
**8**.	2.31 (1.49)	-	.859	2.35 (1.59)	-	.846	2.60 (1.64)	-	.881	3.47 (2.09)	-	.904
**9**.	2.46 (1.61)	.383	-	2.34 (1.62)	.537	-	4.98 (1.57)	.524	-	5.3 (1.34)	.447	-
**10**.	2.39 (1.5)	.732	-	2.56 (1.65)	.692	-	4.42 (1.57)	.741	-	4.72 (1.65)	.751	-
Robust Fit Indices	*χ*^2^ = 58.80, p = .005; CFI = .992; RMSEA = .034; SRMR = .044			*χ*^2^ = 176.25, p < .001; CFI = .990; RMSEA = .037; SRMR = .035			*χ*^2^ = 260.78, p < .001; CFI = .989; RMSEA = .037; SRMR = .035			*χ*^2^ = 71.81, p < .001; CFI = .998; RMSEA = .025; SRMR = .031		
1. I hope that my moral/political beliefs cause other people to want to share those beliefs. 2. I am particularly good at sharing my moral/political beliefs. 3. My moral/political beliefs should be inspiring to others. 4. I often share my moral/political beliefs in the hope of inspiring people to be more passionate about their beliefs. 5. When I share my moral/political beliefs, I do so to show people who disagree with me that I am better than them.							6. I share my moral/political beliefs to make people who disagree with me feel bad 7. When I share my moral/political beliefs, I do so to shame people who do not share those beliefs. 8. When I share my moral/political beliefs, I do so in the hope that people different than me will feel ashamed of their beliefs. 9. I want to be on the right side of history about moral/political issues. 10. Even if expressing my moral/political views does not help anyone, it is important that I share them.					

Upon scoring the newly created sub-scales, correlations revealed support for their hypothesized associates. That is, MGMS:PS was positively, though weakly, correlated with narcissistic extraversion, and MGMS:DS was positively and strongly associated with narcissistic antagonism. Similarly, MGMS:DS was negatively associated with agreeableness, openness, and conscientiousness. These associations are available in Table 3. Full correlations between MGMS subscales and all facet scores of the IPIP-120 and FFNI-60 are available in Supplemental Tables (S2A and S3 Tables), respectively.

**Table 3:** Correlations between the Moral Grandstanding Motivation Scale and key personality variables.

Measure	Study 1		Study 2		Study 3		Study 5	
	MGM: Prestige	MGM: Dominance	MGM: Prestige	MGM: Dominance	MGM: Prestige	MGM: Dominance	MGM: Prestige	MGM: Dominance
Prestige	--	.174**	--	.208**	--	.263**	--	.372**
FFNI: Antagonism	.060	.554**	.122*	.539**	.142**	.599**	.260**	.709**
FFNI: Extroversion	.174**	-.032	.344**	.108	.389**	.333**	.360**	.404**
FFNI: Neuroticism	-.039	-.016	-.103	-.035	-.046	-.063*	.039	.144**
IPIP-NEO Openness	.075	-.227**	.191**	-.127*	.143**	-.155**	-.013	-.348**
IPIP-NEO Conscientiousness	-.017	-.369**	.047	-.241**	.074*	-.353**	-.022	-.493**
IPIP-NEO Extroversion	.087	-.129*	.259**	.014	.374**	.119**	.330**	.175**
IPIP-NEO Agreeableness	.031	-.492**	-.015	-.469**	.005	-.558**	-.082	-.606**
IPIP-NEO Neuroticism	.061	.144*	-.076	.043	-.072*	.227**	.047	.372**
Right Wing Authoritarianism	--	--	--	--	.070*	.176**	--	--
Left-Wing Authoritarianism	--	--	--	--	.020	.267**	--	--

**p < .001;

*p < .05

Comparisons of political ideologies revealed that there were no significant differences between any respondents on MGMS:PS (*F*4,340 = 1.74, *p* = .141). There were significant differences for MGMS:DS (*F*4,340 = 2.72, *p* = .030). However, post-hoc comparisons with Bonferroni corrections revealed no significant differences between political affiliations. With regards to associations with left/right political views, neither MGMS:PS (*r* = -.077; *p* = .177) nor MGMS:DS (*r* = .051; *p* = .372) were significantly associated with political views. However, when a quadratic term was estimated between these variables, a curvilinear relationship was evident for the association between MGMS:PS and Political affiliation (*R*^*2*^ = .045, *F* (2,305) = 7.15, *p* < .001) so that respondents with more extreme answers at either end of the ideological spectrum were higher on MGMS:PS. A similar relationship was not evident for MGMS:DS (*R*^*2*^ = .010, *F* (2,305) = 1.61, *p* = .203).

Both MGMS:DS and MGMS:PS were positively correlated with political and moral conflict. Similarly, in hierarchical regressions, despite such conflict being well-predicted by personality structure (e.g., Big Five Factors and FFNI domain scores), both MGMS:DS and MGMS:PS emerged as positive predictors of greater levels of such conflict, accounting for an additional 5.7% of the variance in the second step of the regression.

Collectively, these results suggest that MG Motivations may be assessed along the hypothesized dimensions of Prestige/Dominance and that they are associated with relevant personality constructs (narcissistic traits; extraversion) and self-reported experiences (i.e., greater political/moral conflict).

## Study 2

In Study 1, a brief measure of MG was developed and initially validated. In order to confirm the structure of the initially validated measure of moral-grandstanding, a second study of undergraduates was conducted.

### Participants and procedure

Study participants were undergraduates in psychology classes at a large, public university in the Midwest. Participants were recruited in Spring of 2018 (*N* = 356; *Mage* = 19.57, *SD* = 2.57; 67.4% women). Sample size was determined by obtaining the largest possible sample in a single semester. Participants predominantly identified as White/Caucasian (78.7%), followed by African-American/Black (15.2%), Asian/Pacific-Islander (3.4%), Latino/Hispanic (2.0%), American-Indian/Native-American/Alaska-Native (1.1%), and Other/Prefer-not-to-say (3%). Participants identified their political parties as being Democrat (34.1%), Republican (24.5%), No Party Affiliation (28.0%), Independent (7.6%), Libertarian (4.8%), and Other (0.9%).

The study procedure was identical to that described in Study 1.

### Measures

Participants completed the same measures in Study 2 as were completed in Study 1. The same 29 item bank for the MGMS was included. However, as the focus of the present work was to confirm the structure of the brief scale, only the 10-item version was scored.

### Plan for analyses

To confirm the factor structure of the 10-item MGMS, we conducted a confirmatory factor analysis with robust diagonally weighted least-squares estimation using the *lavaan* package [63] for R Statistical Software [64].

In keeping with Study 1, we conducted a series of cross-sectional correlational analyses examining the associations between both subscales of the MGMS with personality structure (FFNI and IPIP-120), with ideological affiliation, and with political and moral conflict.

We also conducted hierarchical regression identical to Study 1, wherein the unique contribution of the MGMS subscales to Political/Moral Conflict in daily life was established by first regressing personality traits on such conflict, and, in a subsequent step of the regression, regressing the MGMS subscales on such conflict.

### Results

Confirmatory factor analysis revealed an excellent fit for the factor structure specified in Study 1 (*χ*^2^ = 58.80, *p* = .005; robust statistics: CFI = .992; RMSEA = .034; SRMR = .044).

With regards to political affiliation, neither MGMS:PS (*r* = -.032, *p* = .599) nor MGMS:DS(*r* = -.019, *p* = .758) were associated with left/right ideological leanings. One-way ANOVAs based on political party affiliation revealed no differences between self-identified party affiliation on MGMS:DS (*F*4,305; = 1.99, p = .093). However, significant differences were observed on MGMS:PS (*F*4,305; = 4.79, p = .001), with Bonferroni-corrected post-hoc comparisons revealing self-identified Democrats reporting slightly higher levels of MGMS:PS (*n* = 106; *M* = 4.28; *SD* = 1.09) than self-identified independents (*n* = 24; *M* = 3.61; *SD* = 1.33) and self-identified Libertarians (*n* = 15; *M* = 3.23; *SD* = 1.67).

Subsequent correlations (Table 3) revealed an almost identical pattern of associations to those found in Study 1. Chiefly, MGMS:PS was positively associated with narcissistic extraversion and MGMS:DS was positively associated with narcissistic antagonism. Similarly, MGMS:DS was negatively associated with conscientiousness, agreeableness, and openness. Additionally, in this study, a very weak positive association was found between MGMS:PS and narcissistic antagonism, as well as a moderate association between MGMS:PS and extraversion. Full correlations between MGMS:PS and MGMS:DS and all facet scores of the IPIP-120 and FFNI-60 are available in (S2A and S3 Tables).

With regards to political and moral conflict, both MGMS:PS and MGMS:DS were positively correlated with such conflict (See Table 4). Furthermore, hierarchical regressions revealed that both subscales of the MGMS were uniquely related to conflict, above and beyond the influence of personality alone, accounting for 10.8% of unique variance in Step 2 of the regression (See Table 5).

**Table 4:** Correlations between moral grandstanding and political/moral conflict.

Measure	Study 1		Study 2		Study 3		Study 5	
	MGM: Prestige	MGM: Dominance	MGM: Prestige	MGM: Dominance	MGM: Prestige	MGM: Dominance	MGM: Prestige	MGM: Dominance
Political Moral Conflict	.210**	.339**	.294**	.337**	.351**	.346**	.368**	.630**

** p < .001

**Table 5:** Studies 1–3, 5: Hierarchical regression with standardized estimates (β values) predicting political and moral conflict.

	Study 1		Study 2		Study 3		Study 5	
	Step 1 *β*	Step 2 *β*	Step 1 *β*	Step 2 *β*	Step 1 *β*	Step 2 *β*	Step 1 *β*	Step 2 *β*
FFNI: Antagonism	.316*	.192**	.217*	.087	.142*	.081	.394**	.194*
FFNI: Extroversion	-.001	-.024	.033	-.042	.053	-.022	.058	.026
FFNI: Neuroticism	.028	.032	.048	.044	.013	.025	.012	.020
IPIP-NEO Openness	.066	.080	.165*	.134	.072	.066	-.092*	-.054
IPIP-NEO Conscientiousness	-.178	-.132*	-.030	-.022	-.118*	-.079	-.173**	-.126*
IPIP-NEO Extroversion	-.055	-.035	-.027	-.044	.198**	.112*	.233**	.186**
IPIP-NEO Agreeableness	-.026	-.086	-.076	-.063	-.076	-.102	.100	.032
IPIP-NEO Neuroticism	-.004	.030		.132	.165**	.143*	.244**	.189*
Political View	-.053	-.036		-.029	.014	.019	-	
**MGM: Prestige**		.192**		.242**		.286**		.135**
**MGM: Dominance**		.143*		.241**		.108*		.272**
*R*2	.209	.265	.168	.276	.177	.260	.455	.516
**Δ*R*2**		**.057**		**.108**		**.083**		**.061**
*f*2 for Δ*R*2								
F for Δ*R*2	8.67**	11.33**	5.79**	19.21**	25.20	58.56	58.22	30.76

*p < .01;

**p < .001

Collectively, these results confirm the findings of the first study regarding the measurement of MG Motivations and provide further evidence that MG Motivations are associated with relevant personality constructs and self-reported experiences.

## Study 3

In order to confirm the structure of the MGMS and cross-sectionally validate its utility in a broader-based sample, we conducted a cross-sectional study using a demographically diverse sample matched to U.S. nationally-representative (2010 Census data) demographics.

### Participants and procedure

We obtained a non-probability sample via TurkPrime’s survey panel service [65]. Using this service, a sample (target *N* = 1,000) matching U.S. nationally representative norms for age, gender, U.S. Census region, income, race, and ethnicity was obtained (*N* = 1,063; *Mage* = 48.20, *SD* = 16.38; 49.8% women). Participants were invited to participate in a study titled, “Personality, Beliefs, and Behaviors.” Participants were primarily White/Caucasian (73.8%), followed by African-American/Black (11.4%), Asian-Pacific Islander (8.4%), Latinx/Hispanic (7.1%), American-Indian/Alaska-Native/Native-American (3.2%), and other (1.6%). Participants were free to select whatever racial/ethnic category they believed applied to themselves, including multiple categories. For this reason, total percentages exceeded 100%. Furthermore, recruitment was based on nationally representative norms for race and ethnicity but was separate from these self-identifying questions about race/ethnicity (i.e., Turkprime matched the sample to 2010 norms before such participants completed self-reports of their race/ethnicity). As such, percentages vary slightly from 2010 U.S. Census data. Participants identified their political parties as being Democrat (35.7%), Republican (28.9%), No Party (11.2%), Independent (21.4%), Libertarian (1.8%), and Other (1.1%).

### Measures

The measures used in this study were generally consistent with those used in Studies 1 and 2. Consistent with these prior studies, the entire, randomized 29-item bank for the MG was used, though only the 10 key items of the refined MGMS were scored.

We also included the same measures of political/moral conflict.

We also included two measures of authoritarianism, the Right Wing Authoritarianism Scale [66] and the more recently developed Left Wing Authoritarianism Scale [67].

### Analytic plan

Consistent with Study 2, the primary purpose of the present work was to confirm the factor structure of the refined MGMS in a diverse sample and test its relationships with a variety of key outcomes. The analyses conducted to achieve this goal were consistent with those described in Study 2, with the addition of authoritarianism measures in our trait correlations.

### Results

Consistent with the results of Study 2, CFA (See Table 2) revealed excellent fit for the specified structure (*χ*^2^ = 176.25, p < .001; CFI = .990; RMSEA = .037; SRMR = .035).

Comparisons of political ideologies revealed that there were significant differences between self-identified party affiliation for both MGMS:PS (*F*4, 1,047 = 6.91, *p* < .001) and MGMS:DS (*F*4, 1,047 = 5.01, *p* = .001). Post-hoc comparisons with Bonferroni corrections revealed that, for both sub-scales, the only significant differences were between any identified party affiliations (Democrats, Republicans, Independents, and Libertarians) and those who identified as “no party affiliation,” with identified affiliations all scoring higher than no affiliation. With regards to associations with left/right political views, neither MGMS:PS (*r* = -.002; *p* = .949) nor MGMS:DS (*r* = .039; *p* = .210) were significantly associated with political views. However, when a quadratic term was estimated between these variables, a curvilinear relationship was evident for the association between MGMS:PS and ideological identity (*R*^*2*^ = .051, F (2, 1,060) = 28.67, p < .001) so that respondents with more extreme answers at either end of the ideological spectrum were higher on MGMS:PS. A similar relationship was not evident for MGMS:DS (*R*^*2*^ = .002, F (2,305) = 1.16, p = .313).

Correlational analyses (See Table 3) revealed a similar pattern of findings to both Studies 1 and 2 with regards to personality associates. MGMS:PS was positively associated with narcissistic extraversion and extraversion more generally. MGMS:DS was associated strongly positively correlated with narcissistic antagonism, and negatively associated with agreeableness, conscientiousness, and openness. Additionally, in this sample, MGMS:PS was weakly positively associated with narcissistic antagonism, and MGMS:DS was positively correlated with narcissistic extraversion. Finally, we noted that MGMS:PS was very weakly (*r* = .07, *p* = .023) positively associated with right-wing authoritarianism, while MGMS:DS was weakly-to-moderately correlated with both right- and left-wing authoritarianism. Full correlations between MGMS:PS and MGMS:DS and all facet scores of the FFNI-60 and IPIP-120 are available in Supplemental Tables (S2A and S3 Tables).

With regards to political/moral conflict, both MGMS:PS and MGMS:DS were associated with greater reports of such conflict (See Table 4). Moreover, in regression analyses (See Table 5), both contributed uniquely to such conflict, above the role of personality factors, accounting for 8.3% of the variance in the second step of the regression.

Collectively, findings from Study 3 extend Studies 1 and 2 by replicating their findings regarding the measurement of MG Motivations in a more diverse and representative sample. Additionally, findings from Study 3 suggest that MG Motivations are more closely related to personality constructs associated with status-seeking (i.e., narcissistic traits) than they are related to authoritarianism.

## Study 4

Studies 1–3 established basic links between the MGMS and various relevant constructs. However, to this point, the factor structure of the MGMS had yet to be tested in its constricted form. Therefore, to test the factor structure of only the ten items identified from Studies 1–3, to test basic associations with political ideology, and to establish nationally-representative norms for the scale, a brief, nationally representative sample was collected. The details of this study were pre-registered prior to data collection: https://osf.io/4ev5y/register/5771ca429ad5a1020de2872e.

### Participants

Participants (*N* = 2,000) were recruited via YouGov’s omnibus service [68], and matched to U.S. Nationally Representative (2016 American Community Survey) norms for age, gender, education, income, and race. Sample size was determined via YouGov’s standard omnibus rates. Although YouGov does not utilize probability sampling methods, controlled studies have shown that data obtained by YouGov are often of greater quality and representativeness than competing probability sampling methods [68]. Participants identified their political parties as being Democrat (35.9%), Republican (25.5%), Independent (29.3%), and Other/Unsure (9.2%).

### Measures

Participants completed the ten items of the refined MGMS, as well as two items related to political/moral conflict. These two items both began with the stem, “Over the past 12 months, how often have you:” and measured both negative experiences (e.g., “Experienced conflicts, disagreements, or arguments over political/moral issues with people with views different than your own”) and positive experiences (“Grown closer to other people over political/moral issues”). Responses were recorded on a scale of 1 (*Never/Not at All*) to 8 (*Once a Day or More*).

In addition to these measures, YouGov provided demographic and political variables for each respondent.

Political ideology was measured using YouGov’s supplied ideology question that asked participants to rate their ideology on a scale of 1 to 5, with 1 *(Strongly Liberal*), 3 (*Moderate*), and 5 (*Strongly Conservative*) serving as anchors.

### Results

Consistent with the results of Studies 2 and 3, Confirmatory Factor Analyses (See Table 2) revealed excellent fit for the specified structure (*χ*^2^ = 260.78, p < .001; CFI = .989; RMSEA = .037; SRMR = .035).

Comparisons of political ideologies revealed that there were significant differences between self-identified party affiliation for both MGMS:PS (*F*4, 1,995 = 14.13, *p* < .001) and MGMS:DS (*F*4, 1,995 = 12.79, *p* < .001). Post-hoc comparisons with Bonferroni correction revealed that, for MGMS:PS, Democrats (*n* = 678; *M* = 4.69, *SD* = 1.14) and Republicans (*n* = 471; *M* = 4.81, *SD* = 1.13) scored higher than Independents (*n* = 569; *M* = 4.46, *SD* = 1.13) and those who identified as “not sure” (*n* = 178; *M* = 4.15, *SD* = 1.05). Similarly, for MGMS:DS, Democrats (*n* = 678; *M* = 2.54, *SD* = 1.42) and Republicans (*n* = 471; *M* = 2.45, *SD* = 1.43) both scored higher than Independents (*n* = 569; *M* = 2.20, *SD* = 1.27) and those who identified as “other” (*n* = 103; *M* = 2.24, *SD* = 1.29). Though, for MGMS:DS, those who identified as “not sure” (*n* = 178; *M* = 2.99, *SD* = 1.44) had the highest scores of any group.

Pearson Correlations revealed a very weak, negative association between MGMS:PS and ideology (*r* = -.058, p = .010), showing that more left leaning respondents were slightly more likely to endorse MGMS:PS items. However, there was no observable relationship between MGMS:DS and ideology (*r* = .012; *p* = .402). Curve estimation demonstrated that the relationship between political ideology (Strong Liberal—Strong Conservative) and Prestige Strivings was curvilinear (quadratic) in nature, and accounted for substantive variance in MGMS:PS (*R*^*2*^ = .048; *F*(2, 1787.58) = 44.77, p < .001), so that stronger identification as a liberal or conservative was associated with greater prestige strivings. The relationship between Dominance Strivings and ideology was also curvilinear, though the effect size was extremely small (*R*^*2*^ = .005; *F*(2, 1787.58) = 4.08, p = .017).

Correlation analyses also revealed positive associations between our single item measure of political/moral conflict and both MGMS:PS (*r* = .326; *p* < .001) and MGMS:DS (*r* = .235; *p* < .001). We also found that growing closer to others over moral or political issues was positively associated with both MGMS:PS (*r* = .357; *p* < .001) and MGMS:DS (*r* = .208; *p* < .001).

Collectively, results from Study 4 indicate that the MGMS performs well in nationally representative samples, that MG Motivations are ideologically neutral, while prestige-oriented MG Motivations are associated with polarization. Moreover, these results again demonstrate links between MG Motivations and political/moral conflict.

## Study 5

Collectively, studies 1–4 demonstrated that MG can be reliably measured, that it is associated with narcissistic and domineering aspects of personality, polarized ideology, and political and moral conflicts (above and beyond the influence of personality alone). In order to further examine the utility of the MGMS, we conducted a one-month longitudinal study via Mechanical Turk in an effort to establish MG’s predictive validity in accounting for unique variance in political/moral conflict.

### Participants

Participants for this study were recruited via amazon’s Mechanical Turk via the TurkPrime data-acquisition platform [65]. Participation was restricted to those who 1) resided in the U.S. and 2) had not participated in prior research studies conducted by the first author of this paper. Participants were recruited to participate in a study titled, “Personality and Public Discourse: Mechanical Turk,” which was described as a “25 to 45-minute survey” and for which they were compensated $3.00.

Our enrollment target for the first wave of data collection (baseline data collection) was 500 participants. Such a sample size would allow us to reliably detect cross-sectional associations (e.g., Pearson correlations) of small size (e.g., *r* = .16) with alpha = .05 and power = .95. At baseline, we obtained 499 respondents (47.6% women; *M*_*age*_ = 35.39, *SD* = 10.71). Respondents were predominantly White/Caucasian (79%), followed by African-American/Black (12%), Asian/Pacific Islander (5.6%), Latinx/Hispanic (4.8%), and Other/Prefer-not-to-Say (2%).

Those who successfully completed the first round of the study were contacted again (anonymously via the TurkPrime platform) 30 days later to participate in a brief follow-up study, again for $3.00 in compensation. We aimed for a target enrollment of at least 250 participants (50% retention rate), with no upper-cap on enrollment. A priori estimates of power suggested such a target would supply us with sufficient power (power = .95 at alpha = .05) to reliably detect moderate associations (*r* = .22). Our retention rate was 59% (*n* = 296; *M*_*interval*_ = 30.85 days; *SD* = 1.41).

Multivariate analyses of variance (MANOVA) on key variables (MGMS scales, IPIP 120 scales, and FFNI scores) revealed systematic differences between those who followed up and those who did not. Specifically, those who completed both surveys were more neurotic (*F*[1] = 4.79, *p* = .009), less extraverted (*F*[1] = 5.10, *p* = .003), more agreeable (*F*[1] = 5.47, *p* < .001), more conscientious (*F*[1] = 5.64, *p* < .001), less antagonistic (*F*[1] = 20.10, *p* < .001), lower on FFNI extraversion (*F*[1] = 8.41, *p* < .001), and lower on both MGMS:PS (*F*[1] = 11.69, *p* < .001) and MGMS:DS (*F*[1] = 24.48, *p* = .000). However, when differences in personality structure were accounted for (e.g., when Big Five Factors and FFNI were adjusted for statistically by including them as covariates in the MANOVA), there were no differences in either MGMS:PS (*F*[1] = 2.09, *p* = .171) or MGMS:DS (*F*[1] = 0.70, *p* = .431) between those who followed up and those who did not. Accordingly, personality covariates were included in all longitudinal regression analyses.

### Measures

Measures were generally consistent with prior studies, though we only included the refined, 10-item MGMS (presented in random order) and we also omitted political ideology/affiliation questions from this study. Consistent with prior studies (Studies 1–3), we included the FFNI-60 and the IPIP-NEO-120 as relevant control variables and covariates. We also included the same measure of political/moral conflict as described in Studies 1–3. Finally, we added a single item measuring growing closer to others over moral/political issues. This item read: “Over the past month, how often have you: grown closer to a friend because of your political/moral beliefs?”. Respondents answered on a scale of 1 (*never/not at all*) to 4 (*several times*).

At our one-month follow-up, we included the MGMS scale, and measures of past month political/moral conflict and growing closer to others over moral/political issues.

### Plan for analyses

The analytic plan for baseline (Time 1) data was identical to studies 2–4. Additionally, we computed simple correlations between baseline MGMS scores and one-month (Time 2) scores on the MGMS and political/moral conflict, and growing closer to others over political/moral issues. Finally, two-step hierarchical regressions predicting one-month scores for political/moral conflict and growing closer to others. In the first step of each regression, personality variables (IPIP-120 scales and FFNI scales) and the baseline measure of interest were entered. In the second step of the regressions, baseline MGMS scales were introduced, to determine their unique contribution to key variables over time.

### Results

Consistent with prior studies, CFA with RDWLS estimation revealed excellent fit (See Table 2) for the MGMS as specified (*χ*^2^ = 71.81, p < .001; Robust fit statistics: CFI = .998; RMSEA = .025; SRMR = .031).

Cross-sectional baseline correlations between the MGMS and key variables were consistent with studies 1, 2, and 3 (See Tables 3 & 4), though MGMS:DS was strongly correlated with narcissistic antagonism. Similarly, cross-sectional regressions at baseline were similar to those observed in Studies 1, 2, and 3 (See Tables 5 and 6).

**Table 6:** Study 5, Hierarchical regression with standardized estimates (β values) predicting political moral conflict and growing closer over time.

	Political/Moral Conflict Time 2		Grown Closer Over time	
	Step 1 *β*	Step 2 *β*	Step 1 *β*	Step 2 *β*
FFNI: Antagonism	.001	-.085	.055	-.017
FFNI: Extroversion	.007	-.019	.048	.038
FFNI: Neuroticism	-.04	-.023	-.122	-.107
IPIP-NEO Openness	.017	.042	.091	.103
IPIP-NEO Conscientiousness	-.053	-.017	-.014	-.017
IPIP-NEO Extroversion	.021	.036	-.08	-.107
IPIP-NEO Agreeableness	-.089	-.11	-.1	-.15
IPIP-NEO Neuroticism	-.014	-.027	.035	-.004
Baseline Political/Moral conflict	.646**	.580**	-	-
Baseline: Grown Closer	-	-	.519**	.481**
**MGM: Prestige**		**.044**		.138*
**MGM: Dominance**		.224**		**.071**
*R*2	.480	.515	.331	.354
**Δ*R*2**		**.035**		**.023**
F for Δ*R*2	29.13**	10.33**	15.59**	4.94*

*p < .05;

**p < .001

Over time, the MGMS scale demonstrated relatively good temporal stability (MGMS:PS, *r* = .701, *p* < .001; MGMS:DS, *r* = .663, *p* < .001). Additionally, baseline levels of both scales were associated with political/moral conflict over time (MGMS:PS at T1 with Political/Moral Conflict at T2: *r* = .246, *p* < .001; MGMS:DS at T1 with Political/Moral Conflict at T2: *r* = .484, *p* < .001).

Longitudinal hierarchical regressions were also conducted predicting past-month political/moral conflict and past-month growing closer with others over political/moral issues while controlling for baseline levels of each key variable of interest (See Table 6). In the first step of each regression, baseline levels of the variable of interest (e.g., baseline political/moral conflict in predicting political/moral conflict; baseline recent growth in friendships predicting one-month growth in friendships) emerged as significant and sizable predictors of the same values a month later. For the second step predicting political/moral conflict, MGMS:DS emerged as a significant predictor, contributing an additional 3.5% of unique variance, above and beyond personality controls and baseline levels of the variable of interest. In the second step for growing closer to others over political/moral issues, only MGMS:PS emerged as a significant predictor, accounting for 2.3% of unique variance.

Collectively, the findings of Study 5 suggest that MG Motivations are relatively stable individual difference variables that are uniquely associated with relevant personality constructs, while also uniquely predicting political/moral conflict over time. Moreover, consistent with Study 4, results from Study 5 also suggest that MG Motivations may also predict more positive outcomes over time such as growing closer to others over moral/political issues as well.

## Study 6

In order to establish discriminant validity of the MGMS, above and beyond existing measures of prestige, status seeking, and similar constructs (i.e., social vigilantism), we conducted a final, large-scale study of adults representative of the U.S. population. The study design and hypotheses were pre-registered using the OSF framework (https://osf.io/r8m5k).

### Participants and procedure

To conduct study 6, a U.S. nationally representative sample was collected using YouGov opinion polling. Due to the length of the planned survey, we administered measures in two separate surveys. At baseline, we included measures of grandstanding, political/moral conflict, basic personality, narcissism, general prestige seeking, and social media behaviors. One week later, we administered measures of social vigilantism, status seeking tendencies, and social media behaviors over the past week.

At baseline, we recruited 2,519 adults matched to nationally representative norms for age, gender, race, education, and U.S. Census Region (*M*_*age*_ = 47.5, *SD* = 17.8; 51.4% women; 38.7% Democrat, 27.2% Republican, 25.4% Independent, 3.6% other, and 5.1% not sure; 64.1% White, 12.0% Black, 15.7% Hispanic, 3.3% Asian, 0.9% Native American, 2.5% Mixed, 1.5% other, 0.2% Middle Eastern). One week later, participants were invited to complete additional measures, with a goal of retaining at least 65% of those recruited at baseline. Of those invited, 1,776 (retention rate = 70%) completed measures at 1-week post baseline. Multivariate analysis of variance revealed no systematic differences between those who completed the follow-up and those who did not on the MGMS subscales (Wilk’s λ = .999; *F*(2, 2,751) = 1.77; *p* = .139).

### Measures

#### Moral grandstanding

The Moral Grandstanding Motivation Scale was included with a slight modification. We speculated that the associations between grandstanding and political polarization, as well as its associations with political/moral conflict, could be an artifact of the measurement of grandstanding. Given that each question in the previous administrations of the MGMS referred to “political/moral” beliefs in, the associations between those scale scores and key variables of interest may be inflated (i.e., the use of “political/moral” in each response might naturally select for individuals more prone to engage in political discourse). We attempted to address this by slightly modifying each response. For this study, the words “political/moral” were deleted from each response (i.e., “I hope that my moral/political beliefs cause other people to want to share those beliefs” became “I hope that my beliefs cause other people to want to share those beliefs”). However, we did keep the same stem for the whole scale, which read: “The following items refer to your political or moral beliefs. Take a moment to think about your strongest held moral or political beliefs (it can be both or just one). Think about the issues that are most important to you and the things that you are most passionate about. After thinking about these things, please answer the following questions.”

#### Personality measures

We measured personality using inventories similar to prior works, as well as a few novel measures. Consistent with prior studies, at baseline, we included the FFNI-60 as a measure of narcissistic traits and associated status seeking tendencies. However, to measure the Big Five factors of personality and their associated facets at baseline, we included the SAPA (Synthetic Aperture Personality Assessment) Personality Inventory—81 [SPI-81; 69]. Derived from the larger SAPA Personality Project, the SPI-81 measures the Big Five factors of personality, as well as 27 individual facets.

At baseline, we administered an abridged version of the Prestige subscale of the Dominance-Prestige Scales [34]. Due to time constraints, rather than administering all 17 items of both scales, we only intended to administer the items that the original authors identified as having excellent psychometric properties (6 prestige items and 7 dominance items). However, due to a programming error in survey design, only the 6 prestige items were included in the survey. These 6 items assessing prestige (e.g., “Members of my peer group respect and admire me”) required participants to respond on a scale of 1 (*does not describe me*) to 5 (*describes me very well*). Responses were averaged.

We also included two individual-difference/personality measures at our one-week follow-up. These measures were included at the one-week follow-up rather than the baseline survey due to time constraints (i.e., we could not include all measures at the initial survey due to concerns about length). The first of these measures included in our one-week follow-up was the Social Vigilantism Scale [SVS; 27,28] assessing individuals’ tendency to try to force their beliefs on others or correct the incorrect beliefs of others. In its original form, the SVS required participants to rate their agreement with 14-items (e.g., “I feel as if it is my duty to enlighten other people”) on a scale of 1 (*disagree very strongly*) to 9 (*agree very strongly*). For the present study, we administered these 14 items on a scale of 1 (*disagree very strongly*) to 7 (*agree very strongly*).

The second individual-difference measure included at the one-week follow-up was the Fundamental Social Motives Inventory [56]. This 66-item scale assesses social motivations along 8 dimensions. For the present work, we were only concerned with the status-seeking sub-scale of this measure (example items: “It’s important to me that other people look up to me,” “I want to be in a position of leadership,” and “It’s important to me that others respect my rank or position.”) Participants rated their answers on a scale of 1 (*strongly disagree*) to 7 (*strongly agree*).

#### Political and moral conflict measures

At baseline, we assessed general frequency of political/moral conflict using the same 10 items discussed in earlier studies. We also used the same single item “grown closer to a friend because of my political/moral beliefs” that was used in Study 5.

At baseline, we also included a new 8-item measure of social-media behaviors consistent with grandstanding. This measure required participants to answer the prompt “When was the last time that you…” by rating the recency with which they had engaged in certain behaviors (example items: “Reposted (retweeted/shared) something you disagreed with to make fun of it?” or “Reposted (retweeted/shared) something you agreed with to make yourself look good?”). At baseline, participants responded on a scale of 1 (*never*) to 6 (*within the past 24 hours*).

One week later, we administered this social-media behaviors scale again, changing the prompt (new prompt: “Over the past week, how many times have you…”) and allowing participants to respond on a scale of 1 (*not at all*) to 6 (*more than 10 times*). In both administrations, responses were averaged across items.

#### Analytic plan

Our analyses generally followed our pre-registered plan (https://osf.io/r8m5k), with two slight deviations, as we summarize below.

The factor structure of the MGMS was again confirmed using CFA methods as described in Studies 2–5.

For all key variables, descriptive statistics, internal consistencies, and correlations with MGMS subscales were computed. Correlations between MGMS subscales and facets of both the FFNI and MGMS subscales were also conducted.

Hierarchical regressions were conducted following a similar format to Studies 1, 2, 3, and 5, with the inclusion of social vigilantism, general status seeking, and general prestige seeking as additional predictors in initial steps of the regressions. Given the redundancy of these analyses with the subsequent structural equation models (see below), we report these results in supplemental materials (S4 Table).

**Fig 1:**
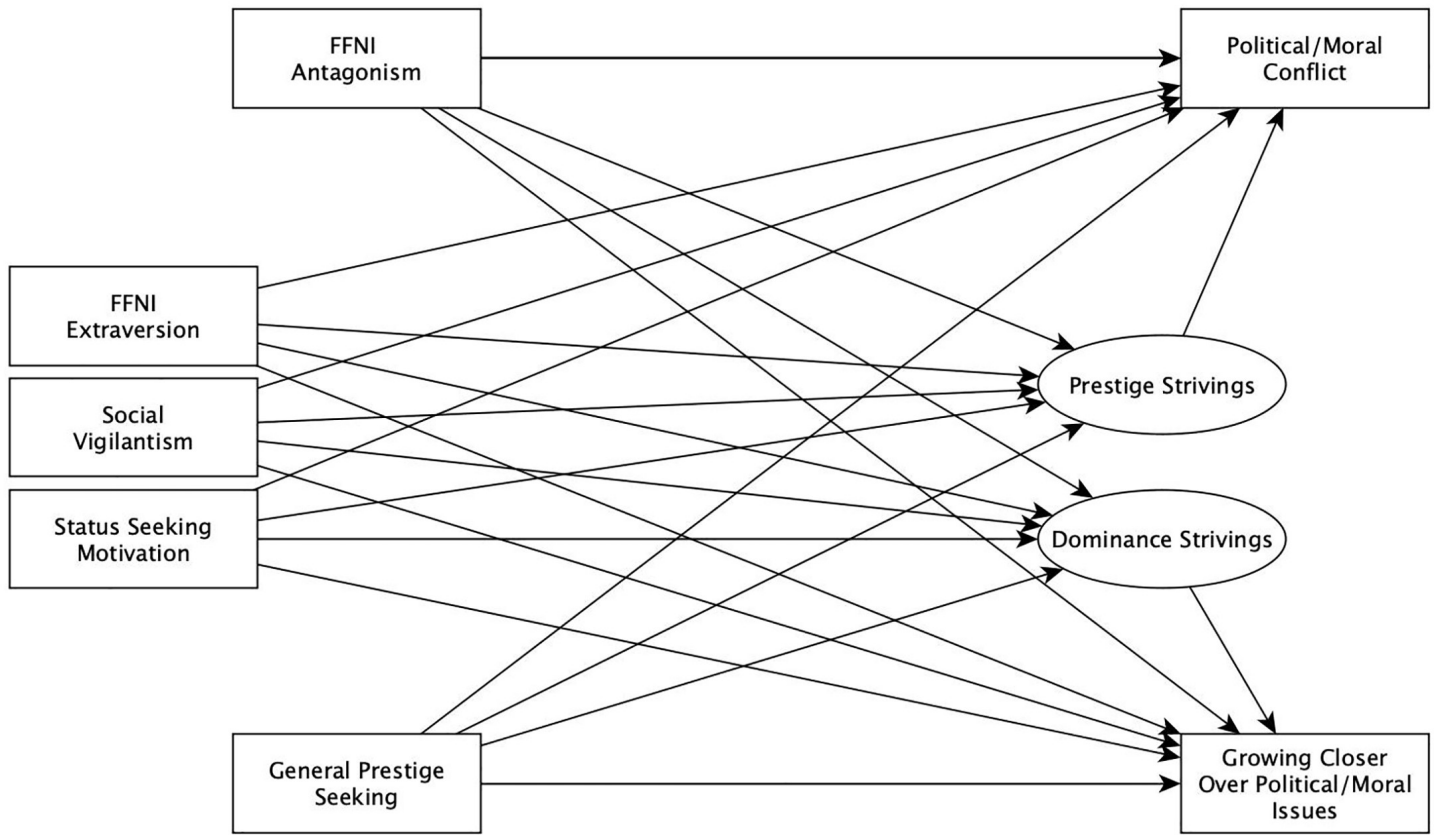
Path diagram illustrating structural equation model predicting growing closer to others and experiencing greater conflict over political/moral issues.

**Fig 2:**
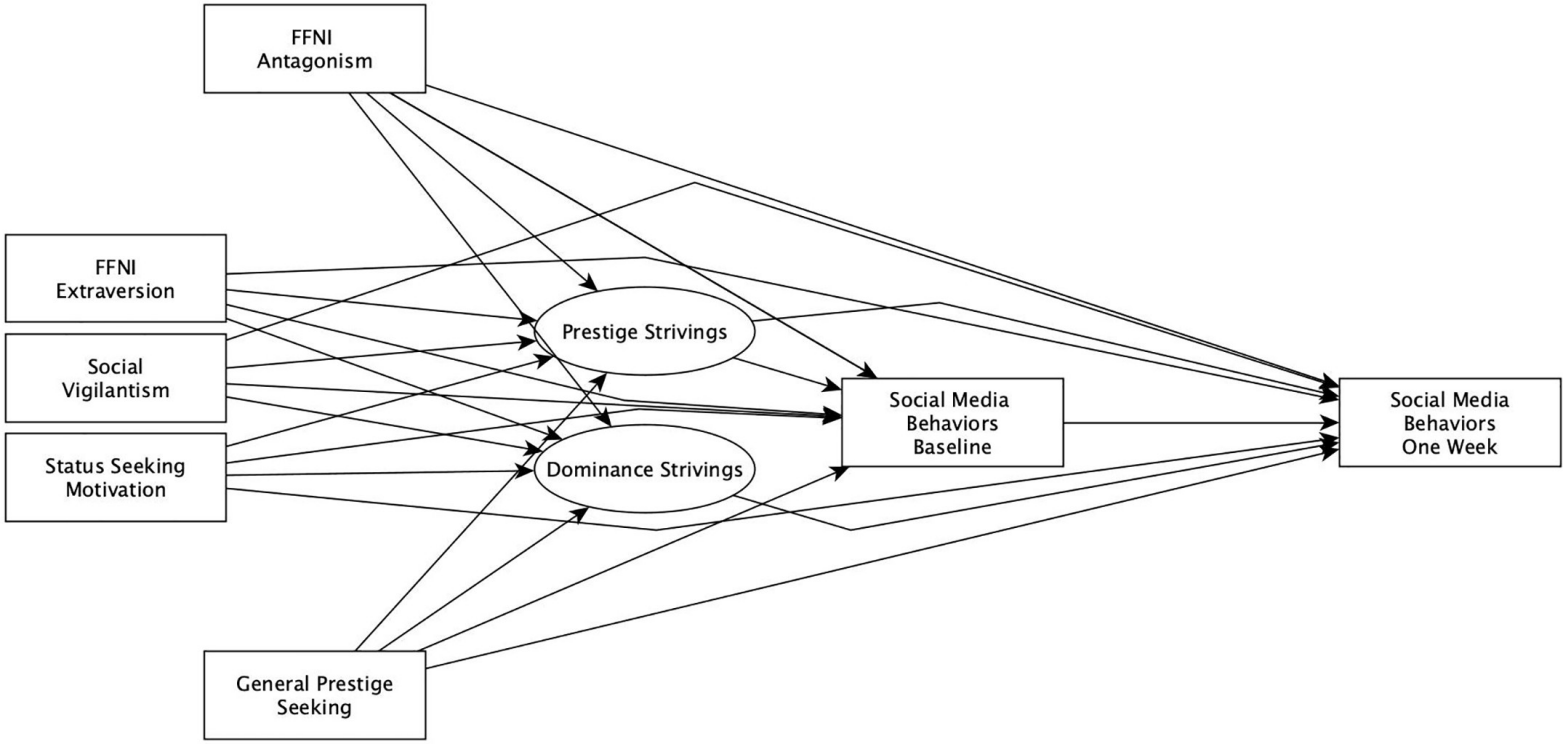
Path diagram illustrating structural equation model predicting social media behaviors at baseline and one week later.

Two Structural Equation Models (SEM) were also conducted. In both, the latent variables *Prestige Strivings* and *Dominance Strivings* were defined by the observed items of the MGMS representing each subscale. Similarly, in both, these latent variables were regressed upon by the observed variables social vigilantism, FFNI Antagonism, FFNI Extraversion, status-seeking motives, and general prestige. In the first SEM, both political/moral conflict and growing closer to a friend over political/moral issues were included as outcome variables, regressed upon by all aforementioned latent and observed variables. This model is summarized in Fig 1. In the second SEM, social media behaviors at baseline were regressed upon by all aforementioned latent and observed variables, and social media behaviors at one week were regressed upon by the same variables, as well as by baseline levels of social media behaviors. This model is summarized in Fig 2.

There were two deviations from our pre-registered analytic plan. Specifically, as we mentioned above, a programming error led to the omission of the dominance items of the Dominance-Prestige scale, which precluded its inclusion in analyses. Secondarily, we mistakenly mis-specified one aspect of our SEM analyses in our pre-registration. In that registration, we noted that the MGMS subscales would be: “…correlated with observed exogenous variables for FFNI Antagonism, FFNI Extraversion, Social Vigilantism, Status-Seeking Dominance, Status-Seeking Prestige, and FSM Status Striving.” However, in our SEM analyses, the MGMS subscales were regressed upon by the listed exogenous variables.

We also conducted the analyses as registered, covarying the specified exogenous variables (i.e., narcissistic antagonism, narcissistic extraversion, social vigilantism, status seeking, prestige seeking) with our latent variables *Dominance Strivings* and *Prestige Strivings*, rather than regressing such exogenous variables on our latent variables. However, this model demonstrated dramatically worse fit (*χ*^2^ = 2003, p < .001; CFI = .777; RMSEA = .120; SRMR = .167) in comparison to the model reported below (Δ*χ*^2^ = 1,908, p < .001).

### Study 6 Results

The MGMS again demonstrated excellent psychometric properties, consistent with prior studies (*χ*^2^ = 85.06, p < .001; CFI = .991; RMSEA = .032; SRMR = .030). Such findings suggest a similar structure of the scale when the “political/moral” phrasing was removed from each response.

Pearson correlations between the MGMS subscales and personality variables followed similar patterns to those seen in studies 1–5 (See Table 7), suggesting the scale performed similarly even with our modified responses. Of note, social vigilantism displayed large (i.e., r ≥ |.30|; [70]) correlations with both MGMS subscales. Both MGMS:PS and MGMS:DS were positively and moderately (i.e., r ≥ |.20|; [70]) associated with status seeking more generally. Additionally, general prestige-seeking was positively and moderately associated with MGMS:PS, while being weakly (i.e., r ≥ |.10|; [70]) and negatively associated with MGMS:DS. Finally, we observed patterns of correlations between relevant personality traits and MGMS subscales that were extremely similar to Studies 1, 2, 3, and 5. Full facet-level correlations between narcissistic traits (FFNI facets) and personality facets (SPI-81 facets) were also similar to prior studies (See S2A, S2B and S3 Tables).

**Table 7:** Descriptive statistics for relevant variables in Study 6 and correlations with Moral Grandstanding Motivations.

Baseline Variables					
	Pos. Range	M (SD)	α	MGMS:PS *r*	MGMS:DS *r*
FFNI Antagonism	1—5	2.24 (0.70)	.881	.162**	.663**
FFNI Extraversion	1—5	2.92 (0.74)	.744	.407**	.303**
FFNI Neuroticism	1—5	2.91 (0.71)	.648	.040*	.125**
SPI: Openness	1—6	4.23 (0.78)	.719	.355**	-.013
SPI: Conscientiousness	1—6	4.14 (0.85)	.775	.101**	-.188**
SPI: Extraversion	1—6	3.55 (0.81)	.768	.241**	.035
SPI: Agreeableness	1—6	4.59 (0.73)	.734	.226**	-.326**
SPI: Neuroticism	1—6	3.44 (1.06)	.832	.072**	.184**
**MGMS: Prestige Strivings**	**1—7**	**4.86 (0.99)**	**.767**	**1**	.163**
**MGMS: Dominance Strivings**	**1—7**	**2.33 (1.45)**	**.908**	.163**	**1**
Political/Moral Conflict	1—4	1.72 (0.71)	.888	.205**	.376**
Grown Closer to Friend	1—4	2.03 (1.11)	--	.224**	.136**
General Prestige Seeking	1—5	3.43 (0.76)	.736	.277**	-.116**
Social Media Behaviors	1—6	2.37 (1.34)	.915	.247**	.270**
**One Week Post-Baseline**					
Social Vigilantism	1—7	4.34 (0.88)	.846	.463**	.297**
Status Seeking Motivation	1—7	3.90 (1.05)	.758	.293**	.278**
Past Week Social Media Behaviors	1—6	1.73 (0.99)	.914	.187**	.346**

*p < .05,

**p < .001

FFNI = Five Factor Narcissism Inventory

SPI = SAPA Personality Inventory

MGMS = Moral Grandstanding Motivation Scale

Pre-registered regression analyses are reported in our supplemental information (S4 Table). Consistent with prior studies, MGMS were associated with greater levels of political/moral conflict, as well as growing closer to others over political/moral issues. In addition, both subscales of the MGMS were positively associated with greater social media behaviors cross-sectionally, and MGMS:DS was positively associated with greater reports of such behaviors at one-week post-baseline. Importantly, in all cases, these relationships were present even when personality covariates (narcissistic traits; Big Five personality characteristics; social vigilantism), fundamental social motives (i.e., status seeking), and general prestige seeking were included as controls.

Finally, results from our pre-registered structural equation models were consistent with hypothesized results. In both models (See Tables 8 and 9), Narcissistic Antagonism emerged as a positive predictor of the latent variable *Dominance Strivings* and a negative predictor of *Prestige Strivings*. In contrast, Narcissistic extraversion emerged as a positive predictor of *Prestige Strivings* but was unrelated to *Dominance Strivings*. Similarly, general prestige seeking was positively predictive of *Prestige Strivings* and negatively predictive of *Dominance Strivings*. Finally, social vigilantism emerged as a positive predictor of both latent variables, but displayed a stronger relationship with *Prestige Strivings*.

**Table 8:** Study 6: Standardized path estimates for structural equation models predicting political/moral conflict and growing closer to friends.

	Dominance Strivings	Prestige Strivings	Political/Moral Conflict	Political/Moral Grown Closer
Prestige Strivings	r = .149**	--	.076*	.094*
Dominance Strivings	--	r = .149**	.273**	.166**
Social Vigilantism	.104**	.418**	.180**	.128**
FFNI Antagonism	.720**	-.145**	.124*	.083
FFNI Extraversion	-.038	.365**	.010	.056
Status Seeking Motivation	-.058	-.035	-.078*	.019
General Prestige Seeking	-.111**	.099**	.021	.139**
R2	.547	.368	.223	.117

*p < .05,

**p < .001

*χ*^2^ = 294.7, p < .001; CFI = .969; RMSEA = .044; SRMR = .032

**Table 9:** Study 6: Standardized path estimates for structural equation models predicting social media behaviors at baseline and one-week later.

	Dominance	Prestige	Social Media Behaviors Baseline	Social Media Behaviors at One Week
Prestige	r = .150**	--	.132**	.048
Dominance	--	r = .150**	.163*	.077*
Social Vigilantism	.105**	.417**	.159**	-.011
FFNI Antagonism	.720**	-.146**	.120*	.149**
FFNI Extraversion	-.038	.367**	.021	-.062*
Status Seeking Motivation	-.035	-.058	-.070*	.150**
General Prestige Seeking	-.110**	.098**	.016	.006
Social Media Behaviors at Baseline	-	-	-	.636**
R2	.548	.368	.163	.517

*p < .05,

**p < .001

*χ*^2^ = 276.36, p < .001; CFI = .972; RMSEA = .043; SRMR = .031

With regards to our outcome variables, in our SEM analyzing the relationships between MG Motivation and political/moral conflict (See Table 8), we found that both *Prestige Strivings* and *Dominance Strivings* were uniquely associated with both greater conflict with others over political/moral issues and greater likelihood of reporting growing closer with friends over political/moral issues (*χ*^2^ = 294.7, p < .001; CFI = .969; RMSEA = .044; SRMR = .032). Notably, these predictive relationships were observed above and beyond the influence of social vigilantism, status-seeking, general prestige, and narcissistic antagonism and extraversion.

In our SEM analyzing the relationships between MG Motivation and social media behaviors (See Table 9), we found that both *Prestige Strivings* and *Dominance Strivings* were uniquely associated with greater levels of such behaviors cross-sectionally (*χ2* = 276.36, p < .001; CFI = .972; RMSEA = .043; SRMR = .031). Notably, these predictive relationships were observed above and beyond the influence of social vigilantism, status-seeking, general prestige, and narcissistic antagonism and extraversion. Additionally, in predicting such behaviors over the past week, *Dominance Strivings* emerged as a positive predictor of such behaviors, even when controlling for baseline levels of social media behaviors.

We also considered several alternative models that were not specified in our pre-registration. For example, we considered a model in which personality traits (i.e., narcissistic traits), status-seeking motives, and general prestige were exogenous variables, with social vigilantism being considered an endogenous variable alongside the latent variables MGMS:PS and MGMS:DS. In this model, both MGMS subscales and Social Vigilantism were regressed upon by FFNI Antagonism, FFNI Extraversion, Status Seeking Motives, and Prestige, and all of the above were then regressed on political/moral conflict and growing closer to others over political moral issues. However, this alternative model demonstrated a worse fit (*χ*^2^ = 451.16, p < .001; CFI = .949; RMSEA = .058; SRMR = .050) in comparison to the pre-registered model reported above (Δ*χ*^2^ = 160.12, p < .001). The same was observed with a similar model predicting social media behaviors, with the alternative model having worse fit (*χ*^2^ = 353.97, p < .001; CFI = .964; RMSEA = .049; SRMR = .040; Δ*χ*^2^ = 160.81, p < .001).

Collectively, these results suggest that MG Motivations exists in the nomological network nearby a variety of similar constructs (Social Vigilantism, Status-Seeking, Prestige, Narcissistic Traits). Importantly, however, MG expands the nomological network, at least in regards to these variables and the others we controlled for in the studies. MG contributed unique variance in the prediction of political/moral conflict and growing closer to others over political/moral issues. Although some of these other traits remained substantive predictors of conflict in discourse (particularly social vigilantism), MG Motivations added incremental validity to the prediction of these outcomes, even over time. Finally, we note that the MGMS performed well, demonstrating strong psychometric properties and virtually identical patterns of correlations to prior studies, even when “political/moral” was deleted from the individual responses.

## General discussion

At the outset of this work, we posited that a newly described phenomena—Moral Grandstanding—is relevant to psychological and social science research about public discourse and polarization. In order to test and validate this construct, six studies were conducted using both cross-sectional and longitudinal designs in university populations, Mechanical Turk workers, and U.S. nationally-representative samples. Below, we seek to summarize these key findings, discuss implications, and propose avenues for future research.

### Measuring Moral Grandstanding Motivations

The primary purpose of the present work was to examine MG as it is described in philosophical literature, contextualize it within social and evolutionary psychologies’ understandings of status-seeking, and develop and validate a scale that measured MG Motivations. We posited that, if MG Motivation is an inherently status-seeking drive, it should be measurable along current paradigms for status-seeking. Across six studies, using diverse sampling methodologies, we found consistent support for this idea and validated a brief measure of this construct (See S5 Table for the final MGMS).

MG Motivation, as presently defined, seems to be measurable in terms consistent with both prestige and dominance strivings. Our ten-item measure of MG Motivation was found to have excellent psychometric properties across a range of politically diverse samples, while also being associated with the personality traits one would expect (e.g., prestige strivings associated with narcissistic extraversion; dominance strivings associated with narcissistic antagonism, both associated with social vigilantism and general status-seeking motivations). Moreover, both subscales of the novel MGMS were consistently (and uniquely) associated with greater conflict in life over moral/political issues, while being only weakly-to-moderately correlated with each other.

Across studies, we also found consistent support for the idea that MG Motivation is ideologically neutral in diverse samples. We consistently found no differences between self-professed Democrats or Republicans on the measure, and most often found no association between either subscale and left-or-right-wing ideology. However, we also found that MG Motivation, particularly Prestige Strivings, is associated with political polarization: At more extreme left-and-right-wing ideological identification, grandstanding motivation was higher. Again, this provides support for the notion that MG is a politically or ideologically neutral construct, while also being clearly associated with polarization of political views. This is consistent with philosophical explorations of the topic, which have previously posited that MG exists independently of political ideology.

We also noted that prestige strivings seem to be more commonly endorsed than dominance strivings across all samples. That is, in each sample, prestige strivings consistently demonstrated a mean value that was at or slightly above the midpoint of the scale (roughly 4 on a 1–7 scale). In contrast, dominance strivings were less frequently endorsed by participants in all studies, consistently averaging more than an entire scale point below the midpoint (e.g., roughly 2.5 on a 1–7 scale). Such findings indicate that people are generally more willing to report engaging in status-seeking via prestige strivings. This is consistent with research on status-seeking, prestige, and dominance more generally [33], which indicates that, while both prestige and dominance are means of seeking status, prestige is more commonly endorsed and engaged in.

### Moral grandstanding and status seeking traits

Across all studies, we found consistent support for the notion that MG Motivation is associated with a general desire to improve one’s status or rank. That is, MG Motivation was associated with various narcissistic traits, which are known to be associated with status seeking [54,71]. Moreover, differing aspects of narcissism (antagonism vs. extraversion) were differentially associated with prestige vs. dominance-oriented MG Motivation. This is again consistent with accounts of narcissism as an indicator for status seeking, as such accounts have noted that the caustic aspects of narcissism (i.e., antagonism, entitlement, rivalry) predict status seeking via more aggressive and dominant methods while the extraverted aspects of narcissism (i.e., admiration and grandiosity) are associated with pursuits of status in pro-social ways [54].

MG Motivations (especially prestige strivings) similarly demonstrated positive associations with Extraversion, which is known to be positively associated with status-seeking. MG Motivations—specifically dominance strivings—were negatively associated with agreeableness, which is consistent with prior work on status-striving more generally. Most importantly, however, in our final study, we demonstrated that MG Motivations are clearly positively related to status-seeking as a discrete construct, and these associations were large (r ≥ .3) according to recent standards [70].

Collectively, these findings provide support for an account of MG that conceptualizes it as a status-seeking behavior that is driven by status-seeking motives.

### Moral grandstanding and public discourse

Collectively, we take this as burgeoning evidence that MG is a potentially useful psychological construct for understanding current problems in public discourse. We believe this work demonstrates that understanding public discourse conflicts in terms of status-seeking and its associated pitfalls may be a useful direction for future work, though we acknowledge that much future research is needed in this domain. That is, the present work is preliminary but promising.

Specifically, MG Motivations are clearly both cross-sectionally and longitudinally associated with greater experiences of moral or political conflict. People who seek status via grandstanding, whether through prestige or dominance strivings, are more likely to experience difficulties in relating to others about moral issues. This is a logical outcome of grandstanding, and it is one that is well-described in philosophical accounts. Moreover, this is likely a byproduct of status-seeking more generally, which is often associated with conflict and discord [72].

Importantly, however, MG Motivation was not the only driving factor in political/moral conflict, as other personality traits and relevant constructs also demonstrated clear associations. For example, in Study 6, social vigilantism was also uniquely associated with general experiences of political/moral conflict and greater endorsement of grandstanding like behaviors both at baseline and one week later. Such a pattern of findings suggests that problems in public discourse are likely attributable to a range of traits and circumstances. The desire to correct or criticize others for having “bad” or “incorrect” beliefs (i.e., social vigilantism) clearly also contributes to such problems. Collectively, this indicates that toxic aspects of discourse are not likely to be easily corrected by simply addressing status-seeking motivations, grandstanding motivations, or any other single cause.

We also note that MG Motivation is associated with reports of having grown closer with others over moral or political issues (Studies 4, 5, and 6). Although the current study design does not allow for definitive causal speculations, such findings are consistent with the idea that MG is a viable mechanism of feeding a status-seeking drive. The desire to seek status within one’s ideological community is likely to result in closer relationships with some individuals within that community even as it may generate conflict and distress in other domains. That is, ultimately, there is likely utility in such nuanced understandings of MG Motivation specifically and polarization in public discourse more broadly. Although conflict and toxic social dynamics are often the foci of popular attention, grandstanding is likely to serve a function for individuals by providing some gains alongside its social costs.

The long-term implications of grandstanding are not yet known, though, should philosophical speculation hold true, it is likely that this phenomenon is associated with polarization and break-downs in effective communication, particularly with outgroup members or ingroup members whom one perceives as a rival. Our findings demonstrate that MG Motivation is likely predictive of greater conflict about moral or political issues over time, but the effects of such conflicts are not immediately evident. That is, although grandstanding is associated with greater reports of having conflict with others about moral and political topics, whether or not these conflicts are distressing, damaging, or unhealthy is not clear. Future work should specifically examine whether or not grandstanding is substantively related to decreases in well-being or increases in loneliness.

We also note that philosophical accounts describe MG as driving polarization. Though our work certainly suggests that MG—specifically prestige seeking—is associated with polarized views, it does not, at this juncture, necessarily point to increases in polarization over time at either the individual or group level. Given our preliminary findings, there is cause to suspect MG could be related to polarization over time, but future work using longer-term longitudinal methods is needed to explore this possibility.

### Limitations and future directions

The present work is the first to specifically examine MG Motivation as a psychological construct. Despite the consistency of our results across diverse samples, we note a few key limitations to the work, as well as several yet unanswered questions. The present work relied primarily on self-report methods which are inherently limited [73]. In some senses, this limitation is likely necessary at this stage of research; starting with self-report is often useful in initial attempts to define and measure a construct. Even so, this leaves the current work relying on individuals’ self-perception and willingness to accurately report on what might be seen as a socially undesirable tendency. There is a need for future research that makes use of diverse means of measurement (e.g., content analyses of written posts and/or multiple informant ratings).

We also note that our studies, while large and diverse, did not assess for several other constructs that may be relevant to MG Motivation (i.e., dogmatism, attitude-resistance, and belief superiority). Taking the example of belief superiority [48,49], or the tendency to view one’s own beliefs as being better than the beliefs of others, this construct bears some similarities to the prestige strivings aspect of MG Motivation. In fact, in some considerations of belief superiority, there is a clear quadratic relationship between polarization and belief superiority [48], similar to the relationship between MG Motivated prestige strivings and polarization. However, we would note that prior works of belief superiority have focused on specific beliefs (e.g., fracking, abortion, healthcare) rather than a global tendency to use one’s own moral beliefs as a cudgel for status-seeking, as MG Motivation does. Even so, future work should specifically examine whether or not MG Motivation is at least a partial mechanism in explaining specific belief superiority.

By focusing on self-report, our methods also limited the measurement and study of MG to the individuals expressing it. As public discourse is inherently a social phenomenon, multiple people are affected, in some way, by others’ engagement. This means that grandstanding likely has effects on other individuals, as well as on the nature and dynamics associated with discourse. This highlights a clear and present need for research that specifically delves into whether or not grandstanding affects discourse, shapes conversation, or changes relational dynamics. For example, it has been hypothesized that MG causes political polarization increases cynicism about the practice of moral discourse, and leads to “outrage exhaustion,” in which one becomes desensitized to public expressions of moral outrage and is unable to muster outrage when it is warranted, due to the overuse of outrage and similar emotions to communicate one’s moral superiority [10]. To establish such effects, however, there needs to be further research that explicitly examines the effects of such speech.

Finally, we note that, despite the representativeness of many of our samples, our research was confined to the U.S., which constrains generality outside of the American cultural context.

## Conclusion

Public discourse regarding morally charged topics is prone to conflict and polarization, particularly on social media platforms that tend to facilitate ideological echo chambers. The present study introduces an interdisciplinary construct called Moral Grandstanding as possible a contributing factor to this phenomenon. MG links various domains of psychology with moral philosophy to describe the use of public moral speech to enhance one’s status or image in the eyes of others. Within the present work, we focused on the motivation to engage in MG. Specifically, MG Motivation is framed as an expression of status-seeking drives in the domain of public discourse. Self-reported motivations underlying grandstanding behaviors seem to be consistent with the construct of status-seeking more broadly, seeming to represent prestige and dominance striving, both of which were found to be associated with greater interpersonal conflict and polarization. These results were consistently replicated in samples of U.S. undergraduates and nationally representative cross-sectional of U.S. residents, and longitudinal studies of adults in the U.S. Collectively, these results suggest that MG Motivation is a useful psychological phenomenon that has potential to aid our understanding of the intraindividual mechanisms driving caustic public discourse.

## Supporting information

S1 Table Study 1, Structure Matrix of Moral grandstanding item bank with maximum likelihood factor analysis with direct oblimin rotation. Extraction Method: Maximum Likelihood; Rotation Method: Oblimin with Kaiser Normalization. ^¥^Item order was randomized across participants. **retained item for final Moral Grandstanding Motivation Scale.
(DOCX)

S2 Table Correlations between IPIP-NEO and SPI-81 facets and the Moral Grandstanding Motivation Scale. **Correlation is significant at the .01 level (2-tailed). *Correlation is significant at the .05 level (2-tailed).
(DOCX)

S3 Table Correlations of Five Factor Narcissism Inventory facets, total score, and grandiose/vulnerable narcissism scales with the Moral Grandstanding Motivation Scale. ** Correlation is significant at the .01 level (2-tailed). * Correlation is significant at the .05 level (2-tailed).
(DOCX)

S4 Table Hierarchical regressions predicting political/moral conflict, growing closer over political/moral issues, social media behaviors, and social media behaviors in the past Week. *p < .05, **p < .005; SPI = SAPA Personality Inventory; FFNI = Five Factor Narcissism Inventory; MGMS = Moral Grandstanding Motivation Scale.
(DOCX)

S5 Table The Moral Grandstanding Motivations Scale. Scoring: Prestige Strivings = Average of items 1–4, 9 & 10. Dominance Strivings = Average of items 5–8.
(DOCX)

S1 Fig Scree plot from Study 1, indicating initial factor structure of Moral Grandstanding Motivation Scale item bank. (TIFF)
