# Supplementary material for: Moral grandstanding in public discourse: Status-seeking motives as a potential explanatory mechanism in predicting conflict
Source: PLoS One. 2019 Oct 16;14(10):e0223749. doi: 10.1371/journal.pone.0223749 (PMC6795490; doi:10.1371/journal.pone.0223749)
Supplement: S1 Table — Extraction Method: Maximum Likelihood; Rotation Method: Oblimin with Kaiser Normalization. ¥Item order was randomized across participants. **retained item for final Moral Grandstanding Motivation Scale. (DOCX) [file pone.0223749.s001.docx]

S1 Table

Study 1, Structure Matrix of Moral Grandstanding Item Bank with Maximum Likelihood Factor Analysis with Direct Oblimin Rotation

| Structure Matrix | | |
| --- | --- | --- |
|  | Factor | |
|  | 1 | 2 |
| 1. ^¥^It is important that others know where I stand on moral/political issues. | **0.765** | -0.284 |
| 1. It is important that I take a stand for what I believe in on moral/political issues. | **0.751** | 0.034 |
| 1. It is important that I take stand for my beliefs on moral/political issues. | **0.747** | 0.094 |
| 1. Being open about my moral/political beliefs is important to me. | **0.740** | -0.119 |
| 1. **I hope that my moral/political beliefs cause other people to want to share those beliefs.**** | **0.759** | -0.096 |
| 1. When I share my moral/political beliefs, I do so to take a stand about what I think is right. | **0.600** | 0.087 |
| 1. **I am particularly good at sharing my moral/political beliefs.**** | **0.653** | **-0.257** |
| 1. I share my moral/political beliefs to let others know that I am an ally. | **0.487** | -0.178 |
| 1. **My moral/political beliefs should be inspiring to others.**** | **0.647** | **-0.261** |
| 1. My moral/political opinions should inspire others. | **0.677** | -0.307 |
| 1. Sharing my moral/political opinions publicly is the best way to let others know where I stand. | **0.659** | -0.27 |
| 1. **I often share my moral/political beliefs in the hope of inspiring people to be more passionate about their beliefs.**** | **0.661** | -0.115 |
| 1. **When I share my moral/political beliefs, I do so to show people who disagree with me that I am better than them.**** | 0.101 | **-0.838** |
| 1. **I share my moral/political beliefs to make people who disagree with me feel bad**** | 0.136 | **-0.824** |
| 1. **When I share my moral/political beliefs, I do so to shame people who do not share those beliefs.**** | 0.134 | **-0.684** |
| 1. **When I share my moral/political beliefs, I do so in the hope that people different than me will feel ashamed of their beliefs.**** | 0.132 | **-0.852** |
| 1. **I want to be on the right side of history about moral/political issues.**** | **0.555** | **-0.02** |
| 1. I want others to know that I am on the right side of history about moral/political issues. | **0.584** | -0.389 |
| 1. If others had insight into moral/political matters like I do, I wouldn’t need to be as vocal about them. | 0.428 | **-0.540** |
| 1. Sometimes, it feels like I am the only one who truly understands moral/political issues. | 0.317 | **-0.551** |
| 1. The more vocal I am about my moral/political views, the more others will be persuaded by them. | **0.534** | **-0.549** |
| 1. When I talk about my moral/political views, I don’t really care what people think of me. | 0.419 | -0.011 |
| 1. If I don’t share my views, the world will be worse off. | 0.423 | **-0.610** |
| 1. Even if sharing my moral/political views alienates people, it is important that I share them. | **0.524** | -0.431 |
| 1. If I don’t share my views, others will be less likely to learn the truth about moral/political matters. | **0.541** | -0.424 |
| 1. I hold moral/political beliefs that I would be embarrassed by if people found out about. | -0.068 | **-0.617** |
| 1. Even if sharing my moral/political views hurts others, it is important that I share them. | **0.617** | -0.383 |
| 1. **Even if expressing my moral/political views does not help anyone, it is important that I share them.**** | **0.657** | -0.307 |
| 1. When it comes to talking about morality or politics, speaking the truth is all that matters. | **0.499** | 0.226 |

Extraction Method: Maximum Likelihood; Rotation Method: Oblimin with Kaiser Normalization.

^¥^Item order was randomized across participants.

**retained item for final Moral Grandstanding Scale
