# Supplementary material for: Moral grandstanding in public discourse: Status-seeking motives as a potential explanatory mechanism in predicting conflict
Source: PLoS One. 2019 Oct 16;14(10):e0223749. doi: 10.1371/journal.pone.0223749 (PMC6795490; doi:10.1371/journal.pone.0223749)
Supplement: S2 Table — **Correlation is significant at the .01 level (2-tailed). *Correlation is significant at the .05 level (2-tailed). (DOCX) [file pone.0223749.s002.docx]

S2 Table A

Correlations between IPIP-NEO facets and the Moral Grandstanding Scale

|  | Study 1  (*N*=361) | | Study 2  (*N*=356) | | Study 3  (*N*=1,063) | | Study 5  (*N*=499) | |
| --- | --- | --- | --- | --- | --- | --- | --- | --- |
|  | **Prestige** | **Dominance** | **Prestige** | **Dominance** | **Prestige** | **Dominance** | **Prestige** | **Dominance** |
| N: Anxiety | -.029 | -.013 | -.082 | -.085 | -.021 | .187** | .094* | .281** |
| N: Anger | -.137** | .208** | .048 | .133* | -.029 | .223** | .073 | .358** |
| N: Depression | .01 | .120* | -.092 | .015 | -.019 | .224** | .063 | .367** |
| N: Self-consciousness | -.038 | .091 | -.120* | -.01 | -.187** | .147** | -.083 | .184** |
| N: Immoderation | .004 | .044 | .036 | .012 | .037 | .102** | .081 | .186** |
| N: Vulnerability | -.054 | .118* | -.139* | .111 | -.153** | .164** | -.033 | .163** |
| E: Friendliness | -.02 | -.108* | .187** | -.004 | .289** | .002 | .292** | .091* |
| E: Gregariousness | .089 | .033 | .116* | .180** | .202** | .144** | .179** | .164** |
| E: Assertiveness | .116* | -.062 | .269** | .014 | .239** | .018 | .195** | .049 |
| E: Activity Level | .015 | -.140** | .184** | -.167** | .220** | .066* | .235** | .092* |
| E: Excitement-Seeking | .111* | -.054 | .141* | .042 | .330** | .319** | .304** | .351** |
| E: Cheerfulness | .110* | -.276** | .150** | -.069 | .343** | -.052 | .298** | .029 |
| O: Imagination | .147** | -.103 | .230** | -.114* | .276** | .152** | .178** | .115* |
| O: Artistic Interests | .084 | -.242** | .160** | -.150** | .172** | -.229** | .036 | -.368** |
| O: Emotionality | -.063 | -.227** | .016 | -.188** | -.024 | -.166** | .025 | -.162** |
| O: Adventurousness | -.005 | .055 | .125* | .079 | .059 | -.065* | -.064 | -.235** |
| O: Intellect | .132* | -.288** | .250** | -.147** | .080** | -.281** | -.071 | -.457** |
| O: Liberalism | .061 | .005 | .097 | .054 | -.052 | .041 | -.128** | -.148** |
| A: Trust | .075 | -.149** | .074 | -.126* | .164** | -.034 | .159** | -.008 |
| A: Morality | -.118* | -.433** | -.099 | -.344** | -.142** | -.572** | -.242** | -.595** |
| A: Altruism | .085 | -.500** | .116* | -.400** | .223** | -.350** | .204** | -.354** |
| A: Cooperation | -.02 | -.414** | -.084 | -.386** | -.130** | -.522** | -.248** | -.680** |
| A: Modesty | -.098 | -.288** | -.123* | -.276** | -.286** | -.465** | -.307** | -.544** |
| A: Sympathy | .192** | -.266** | .121* | -.297** | .243** | -.228** | .193** | -.176** |
| C: Self-Efficacy | .026 | -.301** | .160** | -.194** | .220** | -.146** | .199** | -.134** |
| C: Orderliness | -.025 | -.181** | -.085 | -.135* | .025 | -.188** | -.027 | -.319** |
| C: Dutifulness | .045 | -.460** | .049 | -.381** | .031 | -.493** | -.130** | -.613** |
| C: Achievement Striving | .051 | -.323** | .135* | -.206** | .211** | -.176** | .142** | -.172** |
| C: Self-Discipline | -.165** | -.137** | .056 | -.016 | .018 | -.229** | -.045 | -.344** |
| C: Cautiousness | .014 | -.192** | -.018 | -.124* | -.104** | -.366** | -.166** | -.537** |

** Correlation is significant at the .01 level (2-tailed).

* Correlation is significant at the .05 level (2-tailed).

S2 Table B

Correlations between SPI-81 Facet Scores and the Moral Grandstanding Motivations Scale for Study 6

|  | Prestige Strivings | Dominance Strivings |
| --- | --- | --- |
| SPI81 Compassion | .261^**^ | -.243^**^ |
| SPI81 Irritability | -.006 | .253^**^ |
| SPI81 Sociability | .121^**^ | -.073^**^ |
| SPI81 Well-Being | .042^*^ | -.216^**^ |
| SPI81 Sensation-Seeking | .125^**^ | .413^**^ |
| SPI81 Anxiety | .068^**^ | .109^**^ |
| SPI81 Honesty | .057^**^ | -.544^**^ |
| SPI81 Industry | .029 | -.174^**^ |
| SPI81 Intellect | .126^**^ | -.213^**^ |
| SPI81 Creativity | .316^**^ | -.048^*^ |
| SPI81 Impulsivity | .050^*^ | .360^**^ |
| SPI81 Attention-Seeking | .110^**^ | .199^**^ |
| SPI81 Order | -.013 | -.166^**^ |
| SPI81 Authoritarianism | .062^**^ | -.282^**^ |
| SPI81 Charisma | .282^**^ | .011 |
| SPI81 Trust | .163^**^ | .035 |
| SPI81 Humor | .207^**^ | -.038 |
| SPI81 Emotional Expressiveness | .206^**^ | -.075^**^ |
| SPI81 Art Appreciation | .110^**^ | -.180^**^ |
| SPI81 Introspection | .323^**^ | -.064^**^ |
| SPI81 Perfectionism | .232^**^ | -.014 |
| SPI81 Self-Control | .142^**^ | .059^**^ |
| SPI81 Conformity | .072^**^ | -.180^**^ |
| SPI81 Adaptability | .089^**^ | -.112^**^ |
| SPI81 Easy-going | .094^**^ | -.033 |
| SPI81 Emotional Stability | -.110^**^ | -.005 |
| SPI81 Conservatism | .079^**^ | -.005 |

** Correlation is significant at the 0.01 level (2-tailed).

* Correlation is significant at the 0.05 level (2-tailed).
