# Supplementary material for: Moral grandstanding in public discourse: Status-seeking motives as a potential explanatory mechanism in predicting conflict
Source: PLoS One. 2019 Oct 16;14(10):e0223749. doi: 10.1371/journal.pone.0223749 (PMC6795490; doi:10.1371/journal.pone.0223749)
Supplement: S3 Table — ** Correlation is significant at the .01 level (2-tailed). * Correlation is significant at the .05 level (2-tailed). (DOCX) [file pone.0223749.s003.docx]

S3 Table

*Correlations of Five Factor Narcissism Inventory facets, total score, and grandiose/vulnerable narcissism scales with the Moral Grandstanding Scale*

|  | Study 1  (*N*=361) | | Study 2  (*N*=356) | | Study 3  (*N*=1,063) | | Study 5  (*N* = 499) | | Study 6  (N = 2,519) | |
| --- | --- | --- | --- | --- | --- | --- | --- | --- | --- | --- |
|  | Prestige | Dominance | Prestige | Dominance | Prestige | Dominance | Prestige | Dominance | Prestige | Dominance |
| FFNI Entitlement | .062 | .564** | .099 | .544** | .170** | .579** | .279** | .654** | .146** | .612** |
| FFNI Acclaim-Seeking | .118* | -.274** | .228** | -.177** | .305** | .202** | .241** | .149** | .357** | .169** |
| FFNI Arrogance | .030 | .426** | .126* | .497** | .182** | .554** | .287** | .699** | .179** | .599** |
| FFNI Authoritativeness | .138** | .011 | .289** | .080 | .251** | .104** | .267** | .253** | .350** | .169** |
| FFNI Distrust | .002 | .131* | -.044 | .133* | -.057 | .143** | -.013 | .194** | .021 | .241** |
| FFNI Exhibitionism | .163** | .010 | .231** | .160** | .351** | .240** | .400** | .396** | .305** | .231** |
| FFNI Exploitativeness | .045 | .508** | .075 | .470** | .090** | .566** | .226** | .678** | .098** | .637** |
| FFNI Grandiose Fantasies | .101 | .143** | .270** | .216** | .259** | .441** | .210** | .464** | .214** | .343** |
| FFNI Indifference | .098 | .080 | .110 | .108 | .097** | .108** | .098* | .157** | .059** | .160** |
| FFNI Lack of Empathy | -.085 | .441** | -.038 | .419** | -.059 | .393** | .045 | .529** | -.034 | .532** |
| FFNI Manipulativeness | .040 | .360** | .171** | .294** | .122** | .360** | .246** | .444** | .202** | .395** |
| FFNI Need for Admiration | -.062 | .110* | -.096 | .079 | -.013 | .204** | .104* | .372** | .023 | .285** |
| FFNI Reactive Anger | .060 | .325** | .100 | .235** | .129** | .347** | .219** | .544** | .159** | .433** |
| FFNI Shame | .032 | -.061 | -.050 | -.050 | .003 | .067* | .102* | .172** | .128** | .178** |
| FFNI Thrill Seeking | .116* | .327** | .093 | .285** | .196** | .411** | .275** | .580** | .162** | .443** |
| FFNI TOTAL | .118* | .439** | .227** | .453** | .253** | .569** | .323** | .674** | .269** | .602** |
| Grandiose Narcissism Composite Score | .128* | .418** | .267** | .447** | .289** | .564** | .326** | .635** | .280** | .581** |
| Vulnerable Narcissism Composite Score | .014 | .184** | -.029 | .136* | .024 | .250** | .143** | .433** | .118** | .385** |

** Correlation is significant at the .01 level (2-tailed).

* Correlation is significant at the .05 level (2-tailed).
