# Supplementary material for: Moral grandstanding in public discourse: Status-seeking motives as a potential explanatory mechanism in predicting conflict
Source: PLoS One. 2019 Oct 16;14(10):e0223749. doi: 10.1371/journal.pone.0223749 (PMC6795490; doi:10.1371/journal.pone.0223749)
Supplement: S4 Table — *p < .05, **p < .005; SPI = SAPA Personality Inventory; FFNI = Five Factor Narcissism Inventory; MGMS = Moral Grandstanding Motivation Scale. (DOCX) [file pone.0223749.s004.docx]

S4 Table

Study 6: Hierarchical Regressions Predicting Political/Moral conflict, Growing closer over Political/Moral Issues, Social Media Behaviors, and Social Media Behaviors in the past Week.

|  | Political/Moral Conflict | | Grown Closer | | Social Media Behaviors | | Social Media Behaviors at One Week | |
| --- | --- | --- | --- | --- | --- | --- | --- | --- |
|  | Step 1  *β* | Step 2  *β* | Step 1  *β* | Step 2  *β* | Step 1  *β* | Step 2  *β* | Step 1  *β* | Step 2  *β* |
| FFNI:  Antagonism | .239^**^ | .078^*^ | .023 | -.067 | .200^**^ | .109* | .203^**^ | .161^**^ |
| FFNI:  Extraversion | -.005 | -.013 | .067 | .051 | .013 | -.011 | -.043 | -.048 |
| SPI 81: Openness | .100^**^ | .113^**^ | .002 | .008 | .067^*^ | .072^*^ | -.055 | -.052 |
| SPI 81: Conscientiousness | -.165^**^ | -.161^**^ | -.153^**^ | -.154^**^ | -.157^**^ | -.159^**^ | .025 | .023 |
| SPI 81: Extraversion | .071^*^ | .054^*^ | .056 | .047 | .093^**^ | .085^*^ | .017 | .013 |
| SPI 81: Agreeableness | -.071^*^ | -.093^**^ | .041 | .020 | -.001 | -.028 | .003 | -.006 |
| SPI 81: Neuroticism | .054^*^ | .048^*^ | -.021 | -.027 | .017 | .009 | -.021 | -.023 |
| Social Vigilantism | .206^**^ | .149^**^ | .174^**^ | .127^**^ | .204^**^ | .145^**^ | .031 | .013 |
| Prestige | .027 | .047 | .138^**^ | .147^**^ | .017 | .025 | .001 | .006 |
| Status-Seeking | -.073^*^ | -.061^*^ | .013 | .022 | -.066^*^ | -.056 | -.025 | -.021 |
| Baseline Social Media Behaviors | -- | -- | -- | -- | -- | -- | .655^**^ | .646** |
| **MGMS:**  **Prestige** | **--** | .085^**^ | **--** | **.089**^**^ | **--** | **.122**^**^ | **--** | **.034** |
| **MGMS:**  **Dominance** | **--** | .233^**^ | **--** | **.131**^**^ | **--** | **.132**^**^ | **--** | **.063**^*^ |
| *R*2 | .215 | .249 | .120 | .135 | .161 | .181 | .517 | .520 |
| **Δ*R*2** |  | **.034** |  | **.015** |  | **.020** |  | **.003** |
| F for Δ*R*2 | 49.53 | 40.60 | 21.82 | 15.61 | 35.10 | 22.64 | 171.10 | 5.50 |

*p<.05, **p<.005; SPI = SAPA Personality Inventory; FFNI = Five Factor Narcissism Inventory; MGMS = Moral Grandstanding Motivation Scale
