# Supplementary material for: Moral grandstanding in public discourse: Status-seeking motives as a potential explanatory mechanism in predicting conflict
Source: PLoS One. 2019 Oct 16;14(10):e0223749. doi: 10.1371/journal.pone.0223749 (PMC6795490; doi:10.1371/journal.pone.0223749)
Supplement: S5 Table — Scoring: Prestige Strivings = Average of items 1–4, 9 & 10. Dominance Strivings = Average of items 5–8. (DOCX) [file pone.0223749.s005.docx]

**S5 Table**

**The Moral Grandstanding Motivations Scale.**

| The following items refer to your political **OR** moral beliefs. Take a moment to think about your strongest held moral **OR** political beliefs (it can be both or just one).  **Think about the issues that are most important to you and the things that you are most passionate about.**   After thinking about these things, please answer the following questions: | | | | | | | |
| --- | --- | --- | --- | --- | --- | --- | --- |
|  | Strongly Disagree (1) | Disagree (2) | Somewhat disagree (3) | Neither agree nor disagree (4) | Somewhat agree  (5) | Agree (6) | Strongly agree (7) |
| I hope that my beliefs cause other people to want to share those beliefs. |  |  |  |  |  |  |  |
| I am particularly good at sharing my beliefs. |  |  |  |  |  |  |  |
| My beliefs should be inspiring to others. |  |  |  |  |  |  |  |
| I often share my beliefs in the hope of inspiring people to be more passionate about their beliefs. |  |  |  |  |  |  |  |
| When I share my beliefs, I do so to show people who disagree with me that I am better than them. |  |  |  |  |  |  |  |
| I share my beliefs to make people who disagree with me feel bad. |  |  |  |  |  |  |  |
| When I share my beliefs, I do so to shame people who do not share those beliefs. |  |  |  |  |  |  |  |
| When I share my beliefs, I do so in the hope that people different than me will feel ashamed of their beliefs. |  |  |  |  |  |  |  |
| I want to be on the right side of history about moral/political issues. |  |  |  |  |  |  |  |
| Even if expressing my views does not help anyone, it is important that I share them. |  |  |  |  |  |  |  |

Scoring:

Prestige Strivings = Average of items 1-4, 9 & 10

Dominance Strivings = Average of items 5-8.
